# Addressing Pitfalls in Phase-Amplitude Coupling Analysis with an Extended Modulation Index Toolbox

**DOI:** 10.1007/s12021-020-09487-3

**Published:** 2020-08-26

**Authors:** Gabriela J. Jurkiewicz, Mark J. Hunt, Jarosław Żygierewicz

**Affiliations:** 1 Faculty of Physics, University of Warsaw, L.Pasteura 5 Street, 02-093 Warsaw, Poland; 2 Nencki Institute of Experimental Biology, L.Pasteura 3 Street, 02-093 Warsaw, Poland

**Keywords:** Phase-amplitude coupling, Cross-frequency coupling, Ketamine induced HFO, Olfactory bulb, EEGLAB toolbox

## Abstract

Phase-amplitude coupling (PAC) is proposed to play an essential role in coordinating the processing of information on local and global scales. In recent years, the methods able to reveal trustworthy PAC has gained considerable interest. However, the intrinsic features of some signals can lead to the identification of spurious or waveform-dependent coupling. This prompted us to develop an easily accessible tool that could be used to differentiate spurious from authentic PAC. Here, we propose a new tool for more reliable detection of PAC named the Extended Modulation Index (eMI) based on the classical Modulation Index measure of coupling. eMI is suitable both for continuous and epoched data and allows estimation of the statistical significance of each pair of frequencies for phase and for amplitude in the whole comodulogram in the framework of extreme value statistics. We compared eMI with the reference PAC measures—direct PAC estimator (a modification of Mean Vector Length) and standard Modulation Index. All three methods were tested using computer-simulated data and actual local field potential recordings from freely moving rats. All methods exhibited similar properties in terms of sensitivity and specificity of PAC detection. eMI proved to be more selective in the dimension of frequency for phase. One of the novelty’s offered by eMI is a heuristic algorithm for classification of PAC as *Reliable* or *Ambiguous*. It relies on analysis of the relation between the spectral properties of the signal and the detected coupling. Moreover, eMI generates visualizations that support further evaluation of the coupling properties. It also introduces the concept of the polar phase-histogram to study phase relations of coupled slow and fast oscillations. We discuss the extent to which eMI addresses the known problems of interpreting PAC. The Matlab^®^ toolbox implementing eMI framework, and the two reference PAC estimators is freely available as EEGLAB plugin at https://github.com/GabrielaJurkiewicz/ePAC.

## Introduction

Neuronal activity manifests itself, among others, in the form of periodic voltage fluctuations, which can be recorded as rhythms in electroencephalograms (EEG) and electrocorticograms (ECoG). Conventionally, brain rhythms are studied in discrete frequency bands, which often are attributed to distinct physiological functions (Buzsáki and Draguhn 2004; Wang 2010). Importantly, oscillations of different frequency bands can interact, which may provide a functional mechanism to integrate and organize information across different spatial and time scales (von Stein and Sarnthein 2000; Canolty and Knight 2010).

Phase-amplitude coupling (PAC) is a particular type of cross-frequency coupling (CFC). Previous studies have reported PAC between the phase of a low-frequency oscillation and the amplitude of high-frequency oscillations in local field potentials (LFP) in rats (Lisman and Idiart 1995; Bragin et al. 1995; Tort et al. 2008) and in ECoG and EEG in humans (Canolty et al. 2006; Axmacher et al. 2010; Köster et al. 2014; Demiralp et al. 2007).

PAC has been assigned important functional roles in cognition and neural information processing, specifically, in learning and memory (Lisman 2005; Canolty et al. 2006; Tort et al. 2009; Axmacher et al. 2010), spatial navigation (Tort et al. 2008), sensory signal detection (Händel and Haarmeier 2009), and attentional selection (Schroeder and Lakatos 2009). There is a growing interest in understanding patterns of CFC since they may be relevant for diagnosing and eventually treating various disorders or in designing preventive strategies (Zhang et al. 2016; Berman et al. 2015; de Hemptinne et al. 2013; Koutsoukos et al. 2013).

To estimate coupling between the phase of a low-frequency oscillation and the amplitude of a high-frequency oscillation, these oscillations first need to be extracted from the signal. Most existing methods rely on band-pass filtering combined with the Hilbert transform (Canolty et al. 2006; Tort et al. 2008; Penny et al. 2008). But there are alternative approaches employing wavelet transform (Nakhnikian et al. 2016; Caiola et al. 2019), Reduced Interference Distribution (Munia and Aviyente 2019) or state-space model (Soulat et al. 2019).

The dependence between the phase of a lower-frequency oscillation and the amplitude of a higher-frequency can be evaluated as the clustering of complex vectors, as in method proposed by Canolty et al. (2006). Furthermore, Mormann et al. (2005) proposed measuring the concordance of a time series of phases of a low-frequency oscillation and amplitudes of the high-frequency component. PAC can also be assessed as the measure of non-uniformity of distribution of the mean high-frequency amplitude across the bins of low-frequency phase (Tort et al. 2008) or as the linear model fit measure (Penny et al. 2008). PAC is usually presented in the form of a comodulorgam, i.e., a color-coded map where the color corresponds to the magnitude of coupling, the horizontal axis is the phase-determining frequency, and the vertical axis is the frequency for amplitude.

There are many publications that compare performance of selected existing methods in terms of sensitivity, specificity and robustness to noise (Onslow et al. 2011; Samiee and Baillet 2017; Hülsemann et al. 2019; Tort et al. 2010; Penny et al. 2008; Nakhnikian et al. 2016; Caiola et al. 2019; Soulat et al. 2019). However, they do not indicate one universally optimal PAC measure among the set of comparably well-performing methods with its benefits and drawbacks.

Recently a potential confound that could change the criteria of PAC evaluation was pointed out by Kramer et al. (2008) and van Driel et al. (2015) and Aru et al. (2015). These authors noted that it is important to distinguish PAC, which indeed corresponds to the authentic coupling of two physiological processes, from those that arise as epiphenomena. Physiologically relevant PAC should correspond to the coupling of two separate phenomena and should be a manifestation of the interdependence between them. The spurious PAC can originate from a common source drive, where both low- and high-frequency components are, in fact, coupled with a certain stimulus, either external or internal. Such coupling may arise in experiments involving event-related effects (Voytek et al. 2013).

False detection of coupling in comodulograms can also arise due to a cyclic occurrence, at a frequency corresponding to the low-frequency of the PAC phenomenon, of broadband transient structures (van Driel et al. 2015). These structures do not even have to be oscillatory. They lead to an inhomogeneous distribution of high-frequency amplitude across the phases of the low-frequency cycle detected by most of the currently used methods for the construction of comodulograms. Examples of this effect were given in Gerber et al. (2016). These cyclic processes leading to spurious PACs can also be a manifestation of nonlinear phenomenon, as in the example of the Van der Pol oscillator given by Aru et al. (2015). In this case, the occurrence of PAC is merely another way of expression of the specific shape of the signal, which includes both high and low frequencies in a specific phase arrangement. However, both these low and high-frequency components are, in fact, a manifestation of a particular nonlinear dynamics of the system, not of the coupling of two separate oscillatory processes.

As reported by Aru et al. (2015), and developed in their supplementary literature review, several conditions should be met to indicate meaningful CFC. However, these conditions are not always met in the literature, resulting in a strong over-interpretation of the effects. For now, very few methods confront the problem of spurious PAC detection. One of them is the State-Space PAC method proposed by Soulat et al. (2019), which estimates oscillatory components of the signal directly under the space-state model. This approach eliminates the need for the transition to the frequency domain, which is associated with intricate decomposition of nonlinear structures. Velarde et al. (2019) developed a specialized processing tool (Time-Locked Index) that quantifies the harmonic content of the signal. The harmonically related spectral components associated with a non-sinusoidal waveform may yield a pronounced PAC.

Voytek et al. (2013) offered a partial solution to the analysis of PAC in event-related settings. Also, some of the problems of detection of meaningful coupling were recently addressed by the method of time-resolved phase-amplitude coupling (tPAC) (Samiee and Baillet 2017). These authors included the condition of coexistence of low-frequency oscillation both in the signal for phase and in the signal for high-frequency amplitude and appropriate setting of the bandwidth of the high-frequency band-pass filter.

To address the problem of interpreting PAC and combine most of Aru et al. (2015) recommendations, we propose a comprehensive framework for analyzing this type of CFC, which we have named the *Extended Modulation Index* (eMI). In the following sections, we describe the signal processing steps, statistical methods for controlling the detection of false positives, and graphical presentation of the results. The originality of our approach lies in the automatic PAC origin assessment procedure and integration of auxiliary plots, which supports the interpretation of the coupling phenomenon. We tested the practical use of the eMI tool on a wide variety of simulated data and in vivo electrophysiological data.

## Methods

In this section, we describe the PAC indexes that we will evaluate in “Results”. There are two widely used methods: direct PAC estimator (dPAC) (Özkurt and Schnitzler 2011), and Modulation Index (MI) (Tort et al. 2010), which will serve as reference methods, and the method we propose here – eMI. Further, in this section, we consider a signal *s*(*t*) of duration *T* seconds. The low- and high-frequency oscillations are considered to be present in the same signal, to simplify the description. But, it is possible to use two signals as separate sources of information about low- and high-frequency oscillations. The presumed coupling is between the phase of a low-frequency oscillation ${\Phi }_{f_{P}}$ from the range of phase frequencies *f*_*P*_, and amplitude of a high-frequency oscillation $A_{f_{A}}$. We denote the high-frequencies *f*_*A*_.

### Reference methods

#### Direct PAC estimator

*Mean Vector Length* (MVL) proposed by Canolty et al. (2006), although commonly used, has been shown to be dependent on the absolute amplitude level of the high-frequency oscillation (Tort et al. 2010). The dPAC index circumvents this caveat by including a normalization factor (Özkurt and Schnitzler 2011):

1 $$ \text{dPAC}(f_{P}, f_{A}) = \frac{1}{\sqrt{T}} \frac{\left|{{\sum}_{t=0}^{T} A_{f_{A}}(t) \cdot e^{i{\Phi}_{f_{P}}(t)}}\right|}{\sqrt{{\sum}_{t=0}^{T} A_{f_{A}}(t)^{2}}} \\  $$

where ${\Phi }_{f_{P}}(t)$ is the instantaneous phase of low-frequency oscillation, and $A_{f_{A}}(t)$ is the instantaneous amplitude of high-frequency oscillation. The low- and high-frequency oscillations are obtained by filtering the signal *s*(*t*) around, respectively, low-frequency *f*_*P*_ and high-frequency *f*_*A*_ using EEGLAB toolbox routine *eegfilt.m* which employs a two-way least-squares FIR filter. The order of the filter is equal to the number of samples in three cycles of the corresponding frequency band. The high-frequency filtration bandwidth is equal to twice the maximal *f*_*P*_ in order to capture the spectral effect of coupling. The low-frequency filtration bandwidth is set to Δ*f*_*P*_. To avoid edge effects of filtration, the first and last second of data are excluded from further analysis. The comodulogram is obtained by applying (1) for each pair of frequency for phase *f*_*P*_ and for amplitude *f*_*A*_. In this study, we used the MatlabⓇ implementation provided by the authors in the Supplementary materials (Özkurt and Schnitzler 2011).

#### Modulation Index

*Modulation Index* (MI) proposed by Tort et al. (2010) is also widely used for evaluation of PAC. This measure applies the Kullback–Leibler distance to infer how much an empirical high-frequency amplitude distribution over low-frequency phase bins deviates from the uniform distribution.

For each pair of frequencies for phase and for amplitude (*f*_*P*_, *f*_*A*_) the composite time series $[ {\Phi }_{f_{P}}(t),A_{f_{A}}(t) ] $ is produced. Instantaneous phase ${\Phi }_{f_{P}}(t)$ and amplitude $A_{f_{A}}(t)$ are obtained in an analogous way as in case of dPAC. The only difference is that the first and last second of data after filtration are not excluded from further analysis. The range of phases 〈−*π*,*π*〉 is divided into *J* bins, and the elements of the composite time series are assigned to the corresponding phase bins. The distribution of high-frequency amplitude over low-frequency phase bins is given by:

2 $$ P_{(f_{P}, f_{A})}(j) = \frac{\langle A_{f_{A}}\rangle_{{\Phi}_{f_{P}}}(j)}{{\sum}_{k=1}^{J} \langle A_{f_{A}}\rangle_{{\Phi}_{f_{P}}}(k)}  $$

where $\langle A_{f_{A}}\rangle _{{\Phi }_{f_{P}}}(j)$ is the mean amplitude for phase bin *j*. The distance of this distribution form the uniform one is measured by Kullback–Leibler distance:

3 $$ \text{MI}(f_{P},f_{A}) = \frac{\log(J)+{\sum}_{k=1}^{J} P(k)\log [  P(k) ] }{\log(J)}  $$

The comodulogram is obtained by applying equation (3) to each pair of frequency for phase *f*_*P*_ and amplitude *f*_*A*_. In this study, we used the MatlabⓇ implementation based on Tort et al. (2010).

#### Surrogate data for reference methods

Below, we propose a methodology of producing surrogate data, which is proper both for continuous and epoched data and is suitable to estimate the statistical significance of comodulograms. To generate comodulograms corresponding to data with no-coupling, but with otherwise identical spectral properties, we propose to alter the process of obtaining the instantaneous phase ${\Phi }_{f_{P}}(t)$ for the surrogate data. The surrogate low-frequency oscillation is produced by filtering white Gaussian noise around *f*_*P*_ with the same filters as for extracting the low-frequency oscillations in case of original data. Surrogate comodulograms are obtained by substituting the ${\Phi }_{f_{P}}(t)$ in formulas (1) and (3) with the instantaneous surrogate phase ${\Phi }^{s}_{f_{P}}(t)$.

### Extended Modulation Index Analysis

The eMI analysis is based on the approach introduced by Tort et al. (2010) and recommended by Hülsemann et al. (2019) as it allows detection of a multi-modal coupling. eMI uses the time-frequency (TF) representation of the signal instead of initially proposed filtering to obtain information about the high-frequency oscillations and introduces an automated selection of frequencies for phase exhibiting oscillatory behavior. Moreover, it implements a heuristics for discrimination between reliable and ambiguous couplings.

**Fig. 1:**
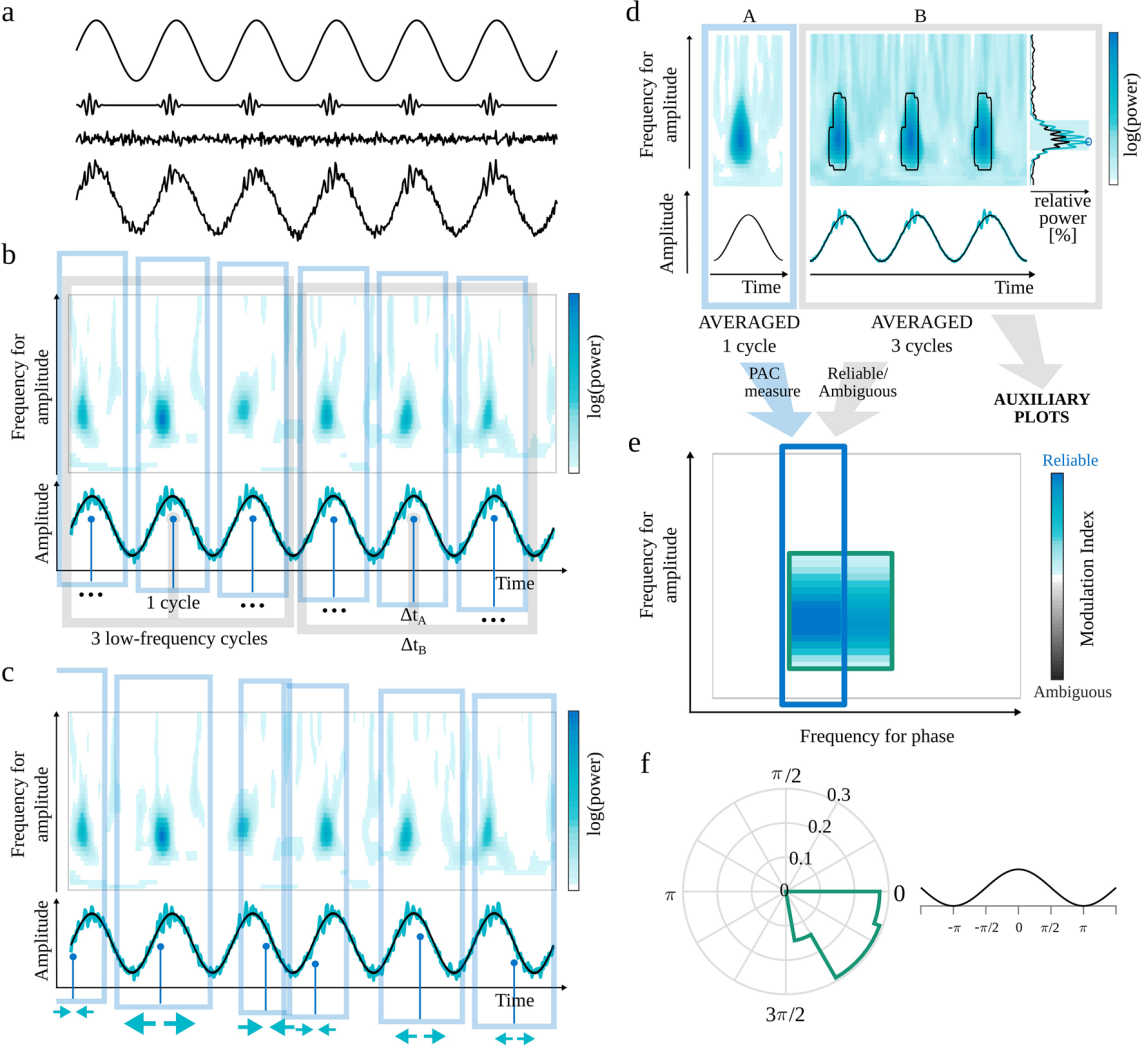
Outline of the eMI method. a) Components of the test signal, from the top: low-frequency oscillation, high-frequency transients, noise and sum of all above. b) Two paths of processing data. Extraction of Δ*t*_*A*_ (1 cycle of low-frequency signal) sections of the *E*(*t*,*f*) map and $s_{f_{P}}(t)$ low-frequency signal (black line) aligned to maxima of $s_{f_{P}}(t)$ indicated by the vertical blue lines. Extraction of Δ*t*_*B*_ (3 cycles of low-frequency signal) sections of the *E*(*t*,*f*) map, $s_{f_{P}}(t)$ low-frequency signal (black line) and *s*(*t*) raw signal (turquoise line) aligned to maxima of $s_{f_{P}}(t)$ indicated by the vertical gray lines. c) Extraction of *randomly* stretched or squeezed Δ*t*_*A*_ sections of the *E*(*t*,*f*) map aligned to *random* phase of the low-frequency signal $s_{f_{P}}(t)$ indicated by the vertical blue lines. d) Two ways of processing data. A—Path leading to calculating PAC measure and building the comodulogram: upper plot—average map $M^{A}_{f_{P}}(t,f)$, lower plot—average extracted low-frequency oscillation $SP^{A}_{f_{P}}(t)$ in black. B—Path resulting in labeling coupling as *Reliable* or *Ambiguous* and producing auxiliary plots for a given low-frequency: upper plot—average map $M^{B}_{f_{P}}(t,f)$ with black outline of regions that produce a statistically significant coupling, lower plot—average signal $S^{B}_{f_{P}}(t)$ in turquoise and average extracted low-frequency oscillation $SP^{B}_{f_{P}}(t)$ in black, right side plot—average spectrum $AS_{f_{P}}(f)$ in black, spectrum of averaged signal $SA_{f_{P}}(f)$ in turquoise, shaded frequency range $\langle f^{max}_{A}-\frac {1}{2}{\Delta } f^{max}_{A},f^{max}_{A}+\frac {1}{2}{\Delta } f^{max}_{A} \rangle $ and $f^{MAX}_{A}$ marked with a blue circle. e) Comodulogram presenting strength of significant coupling and it’s assignment as *Reliable* or *Ambiguous*. Each separate region is outlined with a different color, which acts as a legend for a polar phase histogram. f) Polar phase histogram, which depicts phase histogram for each outlined region from the comodulogram. Values are normalized by the number of all elements in a given area

The eMI toolbox provides additional information on the characteristic of the coupling and addresses most of the recommendations presented in Aru et al. (2015). In the following subsections, we describe the subsequent steps of the procedure. The outline of eMI is illustrated in Fig. 1. Panels a–c depict the key steps of computation and aligning of the original and surrogate time-frequency representations. Panels d–f show the three types of plots, which together inform on the existence and properties of the detected PAC.

#### Obtaining significant low-frequency oscillation

One of the recommendations by Aru et al. (2015) concerned the presence of meaningful oscillations. To ensure the significance of extracted low-frequency, first, we identify low-frequency spectral components that stand out against the background.

For this purpose, we create 200 repetitions of pink noise of the same length as the original signal. For each repetition, we calculate the Welch’s power spectral density estimate.^1^ For each resulting spectrum, we estimate a background level by using piecewise cubic interpolation^2^ between the spectral minima. Next, we produce a distribution of ratios of the pink noise spectrum to the background level for each frequency in the range of frequencies for phase.

The procedure of calculating the spectrum and its background described above is also applied to original data. For each frequency for phase *f*_*P*_, the spectrum to background ratio is obtained and compared with 95^*th*^ percentile of the pink noise spectrum to background ratio distribution. If the original data ratio value is above this threshold, the frequency for phase *f*_*P*_ is labeled as significant, and it undergoes further analysis.

For each significant frequency for phase *f*_*P*_ the low-frequency oscillation is obtained using two-way, zero-phase shift, filtration^3^. The filter was designed as the bandpass Butterworth filter 4^th^ order, with Δ*f*_*P*_ bandwidth.^4^ We will come back to the issue of selecting Δ*f*_*P*_ later. The filtration of *s*(*t*) around *f*_*P*_ yields the low-frequency oscillation $s_{f_{P}}(t)$.

#### Identification of phase

The time positions of the subsequent maxima of $s_{f_{P}}(t)$ are identified. The maxima with a prominence lower than 5% of the median of prominences of all maxima are excluded from further analysis, to focus the investigation only on the meaningful low-frequency oscillations. The subsequent analysis is divided into two paths: (A) leads to measuring PAC and building a comodulogram, and (B) results in labeling given coupling as *Reliable* or *Ambiguous* and producing auxiliary plots (Fig. 1d). The path (A) utilizes sections of the length Δ*t*_*A*_ adjusted for each *f*_*P*_ to contain 1 cycle, while in the second path (B) to contain 3 cycles of the low frequency to analyze the coupling in a broader context. The maxima occurring earlier than $\frac {1}{2}\frac {3}{f_{P}}$ or later than $T-\frac {1}{2}\frac {3}{f_{P}}$ are excluded from further analysis.

Beginning with the first maximum, the section of length Δ*t*_*A*_ centered at this maximum is extracted from $s_{f_{P}}(t)$. Subsequent sections are centered at such consecutive maxima so that the sections do not overlap (Fig. 1b). Afterward, those sections are averaged, yielding the averaged low-frequency oscillation $SP^{A}_{f_{P}}(t)$:

4 $$ SP^{A}_{f_{P}}(t) = \frac{1}{N^{max}_{f_{P}}}{\sum\limits_{n_{f_{P}}=0}^{N^{max}_{f_{P}}} s_{f_{P}}\left( \left[\!t_{n_{f_{P}}}\!-\frac{\Delta t_{A}}{2}\!\right]:\left[\!t_{n_{f_{P}}} + \frac{\Delta t_{A}}{2}\!\right]\right)} $$

where $N^{max}_{f_{P}}$ stands for number of non-overlapping sections centered at consecutive maxima at time $t_{n_{f_{P}}}$ for a given low frequency *f*_*P*_. If $N^{max}_{f_{P}}$ is lower than 3 the analysis for *f*_*P*_ is abandoned. This restriction further supports the requirement of meaningful low-frequency oscillation.

The phase of the averaged low-frequency oscillation $SP^{A}_{f_{P}}(t)$ is computed as the instantaneous phase ${\Phi }_{f_{P}}(t)$ of the analytic signal corresponding to $SP^{A}_{f_{P}}(t)$.

In the path (B), the extraction of subsequent sections of $s_{f_{P}}(t)$ is carried out in the same way but with the length of segments Δ*t*_*B*_. This results in averaged low-frequency oscillation $SP^{B}_{f_{P}}(t)$ which is presented in auxiliary plots (Fig. 1d).

#### Obtaining the time-frequency representation of the signal

We use continuous wavelet transform (CWT) with Morlet wavelets to estimate the energy density of the signal *s*(*t*) in the TF domain (*E*(*t*,*f*)) for a specified range of amplitude frequencies *f*_*A*_ and the whole time *T* (Goupillaud et al. 1984). Caiola et al. (2019) recommendations justify the choice of CWT. For a Morlet wavelet, with the wavenumber, *w*, and translation in time, *u*, the energy density in the time-frequency domain is given by:

5 $$ E(t,f) = \sqrt{\frac{2 \sqrt{\pi} f}{w}} \left| {\int}_{-\infty}^{\infty} s(u) e^{-\frac{1}{2} \left( \frac{2 \pi f(u-t)}{w} \right)^{2}} e^{i2\pi f(u-t)} du \right|^{2}  $$

The presence of edge effects is the issue in the estimation of energy density distributions. In the case of CWT, it can be estimated that these effects will span a time interval equal to the effective support of the wavelet at the lowest frequency on the *E*(*t*,*f*) map on both sides of the analyzed section. To minimize this problem, we cut off fragments of length $\frac {w}{\min \limits (f_{A})}$ distorted by edge effects.

#### Averaging TF map and signal with respect to maxima of low-frequency oscillation

The sections of length Δ*t*_*A*_ are extracted from *E*(*t*,*f*) analogously to “Identification of phase” (Fig. 1b). Those sections are averaged, yielding the $M^{A}_{f_{P}}(t,f)$ map (Fig. 1d):

6 $$ M^{A}_{f_{P}}(t,f) = \frac{1}{N^{max}_{f_{P}}}{\sum\limits_{n_{f_{P}}=0}^{N^{max}_{f_{P}}} E\left( \left[t_{n_{f_{P}}}-\frac{\Delta t_{A}}{2}\right]:\left[t_{n_{f_{P}}}+\frac{\Delta t_{A}}{2}\right],f\right)} $$

In the path (B) the sections of length Δ*t*_*B*_ are extracted from *E*(*t*,*f*) and from *s*(*t*). Those sections are averaged, yielding the $M^{B}_{f_{P}}(t,f)$ map and an averaged raw signal $S^{B}_{f_{P}}(t)$ (Fig. 1d):

7 $$ M^{B}_{f_{P}}(t,f) = \frac{1}{N^{max}_{f_{P}}}{\sum\limits_{n_{f_{P}}=0}^{N^{max}_{f_{P}}} E\left( \left[t_{n_{f_{P}}}-\frac{\Delta t_{B}}{2}\right]:\left[t_{n_{f_{P}}}+\frac{\Delta t_{B}}{2}\right],f\right)} $$

8 $$ S^{B}_{f_{P}}(t) = \frac{1}{N^{max}_{f_{P}}}{\sum\limits_{n_{f_{P}}=0}^{N^{max}_{f_{P}}} s\left( \left[t_{n_{f_{P}}}-\frac{\Delta t_{B}}{2}\right]:\left[t_{n_{f_{P}}}+\frac{\Delta t_{B}}{2}\right]\right)} $$

$M^{B}_{f_{P}}(t,f)$ and $S^{B}_{f_{P}}(t)$ are presented in auxiliary plots (Fig. 1d). They carry additional information that is useful in interpretation of the results.

#### Surrogate data for eMI

Surrogate data should have the same time-frequency structure in the high-frequency range as the original signal, but any potential relation to a low-frequency phase should be removed. To achieve this goal, we altered the process of extracting and aligning sections of the TF maps (Fig. 1c).

The extraction and alignment of Δ*t*_*A*_ sections of *E*(*t*,*f*) is similar as in “Averaging TF map and signal with respect to maxima of low-frequency oscillation” except of two differences. First, the locations are randomly displaced by adding a random value from a uniform distribution (in the range of half of the period of the low-frequency oscillation, i.e., $-\frac {1}{2f_{P}}$ to $+\frac {1}{2f_{P}}$) to the locations of the original maxima. Second, before extracting each section, the map is either squeezed or stretched by a random factor sampled from a uniform distribution 〈0.9,1.1〉^5^. The transformation of the map imitates the variability of the original frequency, *f*_*P*_, due to the nonzero bandwidth of low-frequency.

This step yields one averaged TF surrogate map, denoted $M^{s}_{f_{P}}(t,f)$, for a given low frequency *f*_*P*_. To generate the distribution of possible $M^{s}_{f_{P}}(t,f)$ maps which *a piori* represent no-PAC signals for a given low frequency *f*_*P*_, the above steps are repeated *N*^*s*^ times.

#### Construction of comodulogram

The next step is to quantify the modulation of high-frequency power by low-frequency phase. For each low-frequency *f*_*P*_ the phase ${\Phi }_{f_{P}}(t)$ is obtained (as described in “Identification of phase”). For each high-frequency *f*_*A*_ the power $A_{f_{A}}(t)$ is obtained by $M^{A}_{f_{P}}(t,f_{A})$. The PAC is evaluated by modulation index, utilizing the formulas (2) and (3). The comodulogram is obtained by repeating this operations for each pair of (*f*_*P*_, *f*_*A*_).

The procedure described above is also used to evaluate PAC for surrogate data. The only difference is that the power $A_{f_{A}}(t)$ is obtained by $M^{s}_{f_{P}}(t,f_{A})$. Repeating this operations for each pair of (*f*_*P*_, *f*_*A*_) and each *s* results in *N*^*s*^ surrogate comodulograms. Both original and surrogate *MI* values are centered around mean surrogate *MI* value for each pair of (*f*_*P*_, *f*_*A*_). This operation ensures the comparability between all pairs of (*f*_*P*_, *f*_*A*_) in comodulograms and thus enables us to use the extreme values statistic.

#### Polar phase histogram

For each pair of frequencies (*f*_*P*_, *f*_*A*_), we save the normalized-mean-amplitudes, $P_{(f_{P},f_{A})}(j)$ (2), assigned to phase bins *j*. We also compute the threshold, $th_{(f_{P},f_{A})}(j)$, corresponding to *p*_*PC*_ percentile of the distribution of maximal values of surrogate data normalized-mean-amplitudes. We perform the thresholding for each pair of frequencies (*f*_*P*_, *f*_*A*_) separately. The above threshold values of $P_{(f_{P},f_{A})}(j)$ will be used in creation of auxiliary plots.

For each separate region in comodulogram, the phase histogram is prepared using $P_{(f_{P},f_{A})}(j)$. The count values are normalized by the number of all elements in a given region. The outline color of the phase histogram is consistent with the outline color of the corresponding region in the comodulogram (Fig. 1f).

#### Obtaining average spectrum and spectrum of average signal

A vital part of the heuristics of assessing given coupling as *Reliable* or *Ambiguous* are the average spectrum and the spectrum of an average signal (Fig. 1d). For each section of length Δ*t*_*B*_ extracted from *s*(*t*) (as in “Averaging TF map and signal with respect to maxima of low-frequency oscillation”), the power spectral density is estimated for the whole amplitude-frequency range using periodogram with Blackman-Harris window. Next, all of those spectra are averaged, resulting in the average spectrum, $AS_{f_{P}}(f)$, for each frequency for phase *f*_*P*_. The spectrum of average signal $SA_{f_{P}}(f)$ for each frequency for phase *f*_*P*_ is estimated for average signal $S^{B}_{f_{P}}(t)$, using periodogram with the same parameters as above. Each spectrum is normalized to the total power within the whole amplitude-frequency range.

#### Assignment of Reliable/Ambiguous label

The problem of differentiating coupling with epiphenomenal and proper origins is very complicated. Here, we try to address it, making use of the requirement of the presence of meaningful oscillations proposed by Aru et al. (2015). One of the first steps in the analysis (“Obtaining significant low-frequency oscillation”) ensures the significance of the examined frequency for phase *f*_*P*_. To complete the requirement, we postulate that the statistically significant coupling is reliable when the local maximum in the spectrum within the frequency for amplitude and local maximum in the comodulogram is congruent. In other cases, the coupling should be considered ambiguous. Further, in this subsection, we describe the algorithm implementing this idea.

For each separate region of significant coupling ($\langle {f^{m}_{A}}, {f^{n}_{A}} \rangle $) and for each frequency for phase *f*_*P*_:

- The frequency for amplitude with maximal MI value is determined ($f^{MAX}_{A}$).
- If it is on the lower edge of frequency for amplitude range, it is an uncertain situation, because it is not possible to know if it corresponds to a falling slope of an MI peak in lower frequencies or there is no peak at all. Thus this region is labeled as ambiguous, and a warning is displayed.^6^ In other cases, this region is still a candidate for a reliable coupling.
- Calculate ${\Delta } f^{max}_{A}$—the frequency span (full width at half maximum) of the wavelet envelope at frequency $f^{MAX}_{A}$. The frequency range for a $\langle f^{max}_{A}-\frac {1}{2}{\Delta } f^{max}_{A},f^{max}_{A}+\frac {1}{2}{\Delta } f^{max}_{A} \rangle $ corresponds to the maximum of comodulogram taking into account the frequency resolution of the wavelet. This range is the shaded area in the spectrum in Fig. 1d).
- A local maximum in frequency for amplitude spectrum should be sought within sets union ${f^{M}_{A}} = \langle {f^{m}_{A}}, {f^{n}_{A}} \rangle \cup \langle f^{max}_{A}-\frac {1}{2}{\Delta } f^{max}_{A},f^{max}_{A}+\frac {1}{2}{\Delta } f^{max}_{A} \rangle $.
- It has to be decided which estimate of spectral power to consider for finding a peak, potentially corresponding to the extremum of comodulogram: the average spectrum $AS_{f_{P}}(f)$ or spectrum of average $SA_{f_{P}}(f)$. When the coupled bursts of high-frequency oscillation are strongly synchronized, this high-frequency activity should be more pronounced in the spectrum of average signal than in the average spectrum. Otherwise, the average spectrum is a better choice to look for a peak.
  To find out which type of spectrum contains a more pronounced peak, for each spectrum, the sum of power in frequencies (from ${f^{M}_{A}}$ range) where it exceeds the other spectrum is obtained. The spectrum with a higher sum is used for a search of a maximal power value in frequency range ${f^{M}_{A}}$. The frequency corresponding to this maximum is denoted as $f^{MAX}_{A}$ (marked with a blue circle in spectrum in Fig. 1d). Only proper peaks (with descending slopes on both sides) are considered, hence if the spectrum in $f^{MAX}_{A}$ is a top of a rising slope, the coupling is labeled as ambiguous.
- If $f^{MAX}_{A} \in \langle f^{max}_{A}-\frac {1}{2}{\Delta } f^{max}_{A},f^{max}_{A}+\frac {1}{2}{\Delta } f^{max}_{A} \rangle $ the coupling is labeled *Reliable*. Otherwise, the coupling is labeled *Ambiguous*.

After repeating all steps described above for each frequency for phase *f*_*P*_ and each separate region of significant coupling, the whole comodulogram consists of areas of coupling labeled as *Reliable* or *Ambiguous*. At this point, it is possible to employ one more recommendation presented in Aru et al. (2015)—caution in the presence of harmonics. If there is a harmonic structure in the comodulogram and the coupling for a base frequency is labeled as ambiguous, the rest of harmonics is also labeled as ambiguous.

When the labeling process is done, all *Ambiguous* MI values are presented in the comodulogram in shades of gray. The color of shading of frequency ranges in spectrum $\langle f^{max}_{A}-\frac {1}{2}{\Delta } f^{max}_{A},f^{max}_{A}+\frac {1}{2}{\Delta } f^{max}_{A} \rangle $ also reflects the coupling label. Finally, each separate *Reliable* and *Ambiguous* region in the comodulogram is outlined with a different color (Fig. 1e).

#### Presentation of the results: Comodulogram and Auxiliary plots

The eMI toolbox presents the results of the analysis in three types of plots. The overview is given in the comodulogram (e.g., Fig. 1e), where the statistically significant coupled pairs of frequencies are marked in color (*Reliable*) or shades of gray (*Ambiguous*). The intensity of the color corresponds to the value of MI evaluated according to Eq. 3. Each compact area is outlined with a colored line. The second type of output is the polar phase histogram (e.g., Fig. 1f). It displays phase histogram for each outlined region from the comodulogram—the color in the phase histogram corresponds to the color of the outline of the area in the comodulogram. Values are normalized by the number of all elements in a given area. A fragment of cosine plotted aside serves as an illustration of how a given phase is related to the time course of low-frequency oscillation. Polar phase histograms are useful when investigating phase relations between various areas on the comodulogram. The third type of output is a composite figure (e.g., Fig. 1d, panel B). It is especially useful for the interpretation of PAC. It consists of averaged scalograms covering 3 cycles of the low frequency. The black lines outline areas that correspond to the significant coupling in the comodulogram. Below the map, there are two types of signal: average signal $S^{B}_{f_{P}}(t)$ in turquoise and average low-frequency oscillation $SP^{B}_{f_{P}}(t)$ in black. On the right side, there are two spectra—average spectrum $AS_{f_{P}}(f)$ in black and spectrum of averaged signal $SA_{f_{P}}(f)$ in turquoise. The shaded frequency range $\langle f^{max}_{A}-\frac {1}{2}{\Delta } f^{max}_{A},f^{max}_{A}+\frac {1}{2}{\Delta } f^{max}_{A} \rangle $ shows where the spectral peak should appear to consider it as congruent with the comodulogram. The automatically detected peak in the spectrum, $f^{MAX}_{A}$, which is taken into account while deciding the congruence, is marked with a circle. The composite figures are produced for each significant frequency for phase *f*_*P*_. Besides the figures, the eMI toolbox stores the results in ⋆.mat files for eventual further analysis.

### Statistics

When testing each of the $\left (f_{P}, f_{A} \right )$ pairs of frequencies in a comodulogram, a multiple comparison problem arises. We use the extreme values statistics to take this into account. We generate *N*^*s*^ surrogate comodulograms and select the maximal value from each of them. These extreme values form a distribution. We estimate the threshold corresponding to the *p*_*C*_ percentile from this distribution. The values in the original comodulogram exceeding the threshold *p*_*C*_ indicate a significant PAC in the sense that they are less than $1-\frac {p_{C}}{100}$ likely to be observed in the case of comodulograms of no-PAC signals.

Moreover, we record the corresponding percentile of surrogate data distribution (*P*_*vals*_) for each value in the original comodulogram. Those *P*_*vals*_ allow controlling the False Discovery Rate (FDR) while presenting combined results from more than one time-epoch.

For eMI method to ensure that the coupling corresponds to a reliable augmentation in the time-frequency map, we reject those coupled pairs of frequencies (*f*_*P*_, *f*_*A*_), which do not exceed the $th_{(f_{P},f_{A})}(j)$ threshold for any *j*.

## Materials

In this section, we describe datasets that we used to evaluate the three methods. To test the properties of the eMI method in well-controlled conditions and to compare it with the reference methods, we simulated seven types of signals. Three of them present idealized genuine coupling; the next two are datasets which a priori have no coupling—we use them to test the ratio of false detections. Then we present two models of coupling with epiphenomenal origins. Below we describe all the technical details of the simulations.

Additionally, we examined the performance of all the methods for three physiological sets of data, in which the proper or spurious coupling is expected. Here, the aim was to explore the possibility of discrimination between the physiological and epiphenomenal origins of coupling.

### Models of genuine coupling

The first three models will allow us to test the basic characteristics of the methods. Namely, we will analyze the dependence of the normalized PAC measures obtained for each of the methods on the following parameters:

- *Amplitudes Ratio*—ratio of the high-frequency oscillation amplitude to the low-frequency oscillation amplitude. The default value is 0.1
- *Length Of The Signal* in seconds, the default value equals 10.
- *Noise Level*—ratio of the noise amplitude to the low-frequency oscillation amplitude. The default value is 0.1.

#### Coupled oscillatory bursts

The first model represents a physiologically plausible scenario in which certain phases of a process observed as a low-frequency component promote a process manifesting as high-frequency oscillations. For example, we can imagine the low-frequency process as a gating or clocking mechanism permitting the processing in the high-frequency range. Besides the three general characteristics, i.e., the dependence of the normalized PAC measures on amplitudes ratio, length of the signal, and noise level, this model allows testing *Filling*—ratio of the number of low-frequency cycles with coupled high-frequency bursts to the number of all low-frequency cycles. The default value is 1.

We construct the signal as a low-frequency sine wave with superimposed transients of a high-frequency oscillation occurring in a specific phase of low-frequency oscillation. We set the low-frequency oscillation to 6 Hz, and the high-frequency oscillation to 77 Hz to model commonly measured PAC between theta and gamma rhythms (Lisman and Idiart 1995; Bragin et al. 1995; Canolty et al. 2006; Tort et al. 2008; Köster et al. 2014). The high-frequency bursts are modeled as Gabor function with *σ* = 0.01. The amplitude of the low-frequency oscillation equals 1 and sampling frequency 512 Hz. Additionally, we distort the signal with Gaussian white noise.

#### Amplitude modulation

Amplitude modulation (AM) is a well-known technique used in electronic communication. In this model, the information is coded as the low-frequency variations of the amplitude of the carrier, high-frequency, wave. Here we used the formulation proposed in Tort et al. (2010). The signal consists of a low-frequency sine, mimicking, e.g., theta oscillation, a high-frequency sine, imitating, e.g., gamma band, with amplitude altering according to the phase of the low-frequency sine, and white noise. Besides the standard characteristics, this model allows us to examine the influence of the depth of modulation. The amplitude of high-frequency oscillation $A_{f_{A}}$ is modulated as:

9 $$ A_{f_{A}}(t) = \overline{A_{f_{A}}} \frac{(1-\chi) sin(2 \pi f_{P} t) + 1 + \chi}{2} $$

where $\overline {A_{f_{A}}}$ is constant determining the maximal amplitude of high-frequency oscillation and *χ* ∈ [0,1] is the fraction of the $A_{f_{A}}$ that is not modulated by *f*_*P*_; *f*_*P*_ is the frequency of the slow oscillation, here set to 6 Hz; *f*_*A*_ is the frequency of the fast oscillation, here set to 77 Hz.

The depth of modulation is defined as 1 − *χ*, the fraction of the $A_{f_{A}}$ that is modulated by *f*_*P*_. Default value of *χ* is set to 0.1. The modeled signal is:

10 $$ s(t) = A_{f_{A}}(t) sin(2 \pi f_{A} t) + \overline{A_{f_{P}}}sin(2 \pi f_{P} t) + \overline{A_{N}} W(t)  $$

where $\overline {A_{f_{P}}}$ is a constant determining the amplitude of low-frequency oscillation (here, set to 1), *W*(*t*) is Gaussian white noise derived from the standard normal distribution, $\overline {A_{N}} \in [ 0,1 ] $ is the amplitude for white noise. The sampling frequency is 512 Hz.

#### Multimodal coupling

Some of the PAC methods have problems detecting the coupling if it occurs at multiple phases of the slow oscillation. To evaluate this characteristic, we simulated signals consisting of high-frequency sine coupled with multiple phases of low-frequency oscillation, with the controlled parameter *Number of modes* (Tort et al. 2010). This model employs formula (10) but with a different definition of $A_{f_{A}}$:

11) (12 $$ \begin{array}{@{}rcl@{}} A_{f_{A}}(t) &=& \overline{A_{f_{A}}} [  (1-\chi) \sum\limits_{m=1}^{M}{g_{m}(f_{P},t)} + \chi ]  \\ g_{m}(f_{P},t) &=& \frac{G [  sw(f_{P},t) ]  - min [  G(sw) ] }{max [  G(sw) ]  - min [  G(sw) ] } \end{array} $$

where *M* is number of phase modes, *G* denotes a normal distribution function with zero mean and variance *σ*^2^ = 0.1 and *sw*(*f*_*P*_, *t*) is a sawtooth wave of frequency *f*_*P*_. Different modes are obtained by presenting different phase lags in the sawtooth wave.

The rest of the parameters are set to default values. The sampling frequency is 512 Hz.

### Signals for testing false alarm ratio

To validate the process of surrogates generation and the effectiveness of the false alarm control, we simulated two types of signals which a priori have no coupling.

#### Random oscillatory bursts

This signal is generated as a no-coupling counterpart to the coupled oscillatory bursts (“Coupled oscillatory bursts”). However, here transients of fast oscillation appear in random phases of the slow oscillation. We examine the dependence of the PAC estimators on *Noise Level*. The signal length is 10 seconds, and the sampling frequency is 512 Hz. The other parameters are set to default values.

#### Filtered noise

These simulations are meant to be a no-coupling counterpart to the AM coupling model. The signal consists of low-frequency sine (*f*_*P*_ = 6 Hz, amplitude set to 1) and high-frequency oscillation obtained by band-pass filtering Gaussian white noise with cutoff frequencies [76,78] Hz (Butterworth 2^nd^ order). The amplitude of resulting high-frequency oscillation is normalized so that the maximum is at 0.1. This signal is embedded in white noise derived from the standard normal distribution. We examine the impact of *Noise Level* on the PAC measures. The signal length is 10 seconds, and the sampling frequency is 512 Hz.

### Models of waveform-dependent coupling

Both periodic and non-periodic sharp waveforms may produce PAC (Gerber et al. 2016). To test the ability to detect and identify as ambiguous a coupling which arises due to the shape of the signal, we used two signals containing a series of Gaussian functions.

#### Periodic trains of Gaussian functions

This signal is a replication of the one proposed by Gerber et al. (2016). It is used to demonstrate how a semi-periodic occurrence of sharp waveforms can yield spurious coupling. In this case, the slow-rhythm is produced by a train of Gaussian-shaped spikes placed at intervals drawn from a uniform distribution of 100 ± 20 ms, which corresponds to spectral peak at 10 Hz. This signal is added to an EEG signal that does not contain any PAC. The Gaussian spikes amplitude is set to either 2 or 5 standard deviations of the background EEG signal. The full-width-at-half-maximum of the Gaussian spikes is set to 15 ms. The sampling frequency is 1000 Hz. The preprocessing included highpass filtering with cutoff frequency 1 Hz (Butterworth 2^nd^ order), lowpass filtering with cutoff frequency 250 Hz (Butterworth 2^nd^ order) and selecting from this signal a 10 s fragment.

#### Non-periodic trains of Gaussian functions

This signal is used to demonstrate that periodicity is not strictly necessary for PAC as long as the sharp waveforms are spaced sufficiently apart for a given frequency-for-phase such that the peaks of this slow frequency can align with the sharp waves (Gerber et al. 2016). This signal is a non-periodic equivalent of the signal described above as a periodic case. Both signals contain the same average count of sharp waveforms in the form of Gaussian functions with the same width. This non-periodic version was obtained in the following way. A series of 100 events was obtained as random time values sampled uniformly from an interval 0 to 10 s with 0.001 s step. A Gaussian spike of width 15 ms is placed at each event, and the resulting signal is superimposed with a 10 s background EEG signal that does not contain any PAC (we used the same EEG signal as in periodic trains of Gaussian functions, “Periodic trains of Gaussian functions”). The Gaussian spike amplitude is set to 2 or 5 standard deviations of the background EEG signal. The sampling frequency is 1000 Hz. The preprocessing included highpass filtering with cutoff frequency 1 Hz (Butterworth 2^nd^ order) and lowpass filtering with cutoff frequency 250 Hz (Butterworth 2^nd^ order). Both filters were utilized on a whole EEG signal with Gaussian spikes added, before the extraction of the 10 s fragment.

### Real data

We also performed analysis on a real in vivo data. We chose examples of both epiphenomenal and physiological coupling origins. The in vivo data consists of:

- recording from a rat with epilepsy, which contains electrographic seizures. We predict (based on Samiee et al. (2018)) that this type of signal should produce PAC. Additionally, it is postulated that the periodic seizure sharp transients are generated by a dynamic nonlinear process (Pijn et al. 1997), which motivates us to assume that it could be a waveform dependent (spurious) coupling.
- mu rhythm in ECoG signal, which is reported to produce PAC (Gerber et al. 2016). It contains a periodic series of sharp transients, and with this characteristic waveform, we associate the epiphenomenal origins of the coupling.
- recordings from rats after ketamine injection. This type of data is reported to produce PAC (Cordon et al. 2015; Pittman-Polletta et al. 2018; Caixeta et al. 2013; Ye et al. 2018). During visual inspection of the raw signal, there are visible distinct low- and high-frequency oscillations. The presence of distinct oscillations suggests the physiological origins of a potential coupling.

#### Recording from a rat with epilepsy

This in vivo data come from Meeren et al. (2002) from one rat of the inbred WAG/Rij strain. This strain suffer from a genetically determined seizure disorder in which rats experience hundreds of spontaneous electrographic seizures a day.

The rat was implanted with two rows of seven electrodes (stainless steel wires of 100 microns diameter, insulated except at the tip, California Fine Wire, Grover Beach, CA) in rostrocaudal direction over the dorsal aspect of the cortical surface, one row in each hemisphere (AP=[+ 4.0,+ 2.0,+ 0.0,-2.0,-4.0,-6.0,-8.0], ML=± 3.0 mm with respect to the bregma). The electrode placed at (AP=+ 2.0, ML=-3.0 mm) was selected for further analysis.

Ten days after the surgery local field potentials were recorded through a multichannel differential amplifier (DATAQ Instruments, Inc., Akron, OH), bandpass filtered between 1 and 1000 Hz, digitized with 2000 samples per second. The rat was recorded in the freely moving state until enough representative spontaneously occurring spike-wave discharges were collected. All experiments were approved by an Ethical Committee on Animal Experimentation of the University of Nijmegen.

Preprocessing of the in vivo data included highpass filtering with cutoff frequency 1 Hz (Butterworth 2^nd^ order), lowpass filtering with cutoff frequency 250 Hz (Butterworth 2^nd^ order) and selecting from the recording 10 s fragment containing epileptic seizure.

#### Mu rhythm in ECoG signal

We tested one of the cases of intracranial electroencephalographic recordings (ECoG) reported in (Gerber et al. 2016) to produce waveform-dependent CFC. These authors made the data available as supplementary materials. We used the data containing mu-oscillations, which were obtained from a patient performing a task that involved viewing images from several categories and responding by a button press to a target category. The electrode included in this study was located on the right primary somatosensory cortex of the patient.^7^

Preprocessing of the data included highpass filtering with cutoff frequency 1 Hz (Butterworth 2^nd^ order), lowpass filtering with cutoff frequency 250 Hz (Butterworth 2^nd^ order) and selecting from the recording 10 s fragment containing pronounced mu rhythm.

#### Recordings from rats after ketamine injection

This in vivo data come from Hunt et al. (2019). Seven male Wistar rats (220-250 g) were implanted with twisted tungsten wire electrodes (125 microns, Science Products, Germany), insulated except at the tip, bilaterally in the olfactory bulb (AP=+ 7.5, ML=± 0.5, DV= 3-3.5 mm, with respect to the bregma) and unilaterally in the ventral striatum (AP=+ 1.6, ML=+ 0.8, DV= 7 mm). One week after surgery local field potentials were recorded through a JFET preamplifier, amplified x1000, filtered 0.01–1000 Hz and digitized at 1.6 kHz. Rats were recorded in the freely moving state. They were habituated to the recording chamber over 2 days, approximately 20 minutes per day. On the experimental day baseline LFP’s were recorded for approximately 20 min before, and up to 60 min post intraperitoneal injection of 25 mg/kg ketamine.

At the end of the study brains were sectioned (40 microns) using a cryostat and mounted on gelatin-coated microscope slides. The location of the electrode tips was determined on Cresyl violet stained sections.

All necessary measures were taken to minimize pain or discomfort and the number of experimental animals used in this study. All experiments were conducted in accordance with the European community guidelines on the Care and Use of Laboratory Animals (86/609/EEC) and approved by a local ethics committee.

Preprocessing of the real data included highpass filtering with cutoff frequency 0.1 Hz (Butterworth 2^nd^ order), lowpass filtering with cutoff frequency 230 Hz (Butterworth 2^nd^ order), downsampling to 465 Hz and dividing data into 20 s fragments. The epochs containing artefacts were excluded from the analysis. The epochs corrupted with improbable data or with abnormally distributed data were marked automatically as artefacts using the EEGLAB routines *pop_jointprob.m* (with probability of activity limit in terms of standard deviation set to 5) and *pop_rejkurt.m* (with activity kurtosis threshold in terms of standard deviation set to 5), respectively.

## Results

**Fig. 2:**
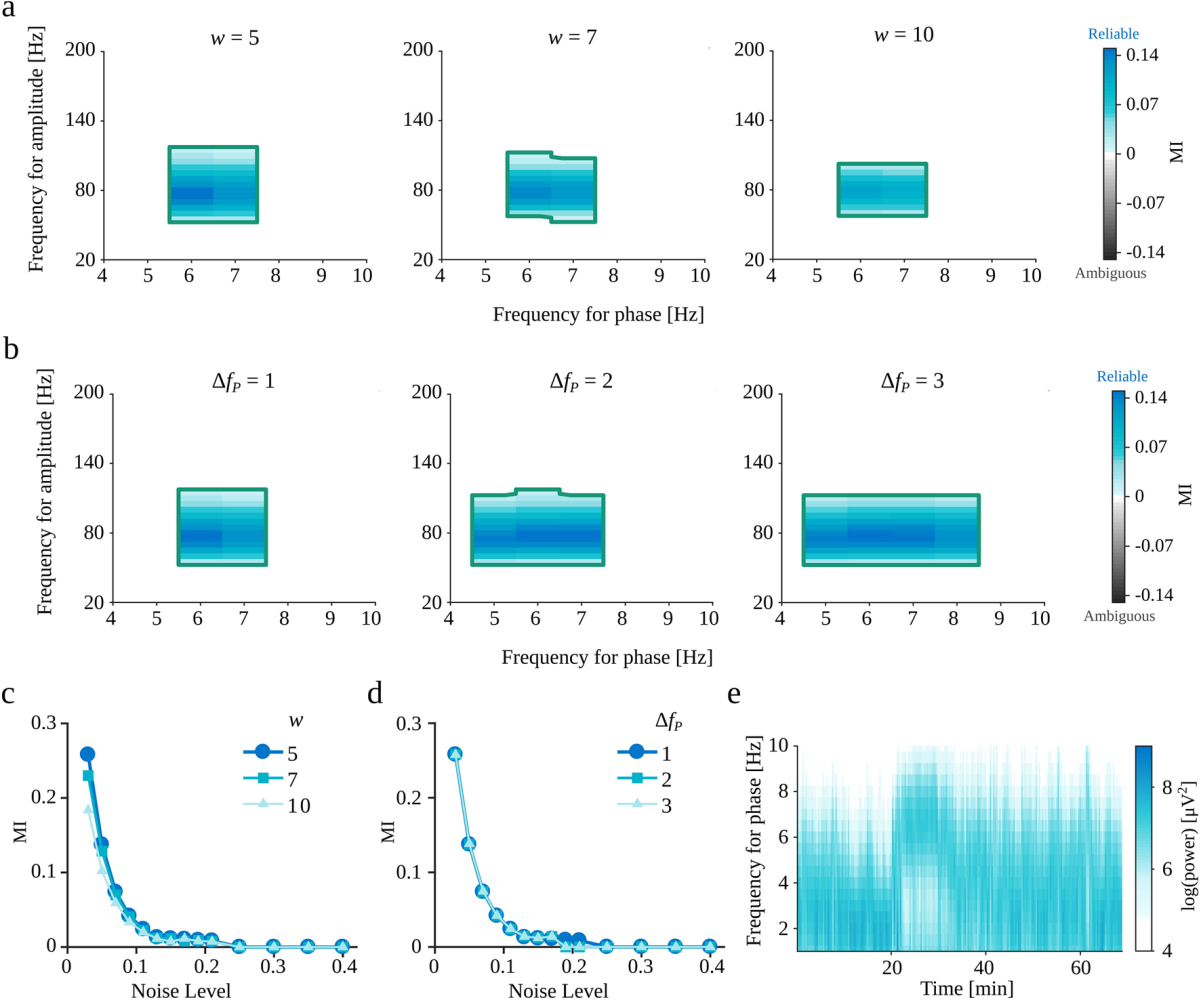
**a–d** Dependence of eMI results on the choice of parameters *w* and Δ*f*_*P*_ for coupled oscillatory burst signal (“Coupled oscillatory bursts”) generated with *Noise Level*= 0.05 and other parameters set to default values. **a** Comodulograms for *w* ∈{5,7,10} and Δ*f*_*P*_ = 1. **b** Comodulograms for *w* = 5 and Δ*f*_*P*_ ∈{1,2,3}. **c–d** Dependence of MI value for the coupled pair of frequencies on the *Noise Level* and *w* (**c**) and Δ*f*_*P*_ (**d**). Marks indicate average across 10 repetitions. **e** Spectrogram of recording from exemplary rat after ketamine injection (“Recordings from rats after ketamine injection”) for a range of phase frequencies *f*_*P*_. The width of the frequency range of visible activity (after ketamine injection around 20 min) suggests the choice of parameter Δ*f*_*P*_= 2 Hz

The eMI toolbox requires setting two hyperparameters, i.e., the wavelet wave number *w* and the bandwidth of the low-frequency filter Δ*f*_*P*_. Illustratory considerations are shown in Fig. 2. As can be seen in panels c and d, the value of MI measure essentially does not depend on the choice made. The selected parameters influence mainly the size and shape of the *f*_*P*_ − *f*_*A*_ space displaying reliable coupling. In line with prediction, along with the increase of a wavelet wave number *w* there is a decrease in time resolution and increase in the frequency resolution. Hence the coupled *f*_*A*_ region decreases. Whereas the increase of the Δ*f*_*P*_ results in decrease in specificity in frequency for phase.

There are some guidelines concerning the choice of parameters. We should choose the wavelet wave number *w* so that its time span for a presumed high-frequency is not bigger than duration of a one presumed low-frequency cycle. It provides a better fit to the signal characteristic. Whereas the bandwidth of the low-frequency filter Δ*f*_*P*_ should be adjusted to the spectral features of a low-frequency oscillation by visual inspection of a spectrum in a frequency for phase range. For highly variable low-frequency oscillations, wider bandwidth is a better choice. An example of a spectral features inspection is a spectrogram of the recording from rat after ketamine injection (“Recordings from rats after ketamine injection”) presented in Fig. 2e, which displays a wide-band activity with a 2-3 Hz width of a frequency range.

For all analysis the number of phase bins was set to 18 following (Tort et al. 2010). The number of repetitions while producing surrogate data *N*^*s*^ was set to 200, and percentiles *p*_*C*_ as well as *p*_*PC*_ were set to 95.

### False Positive Ratio

**Fig. 3:**
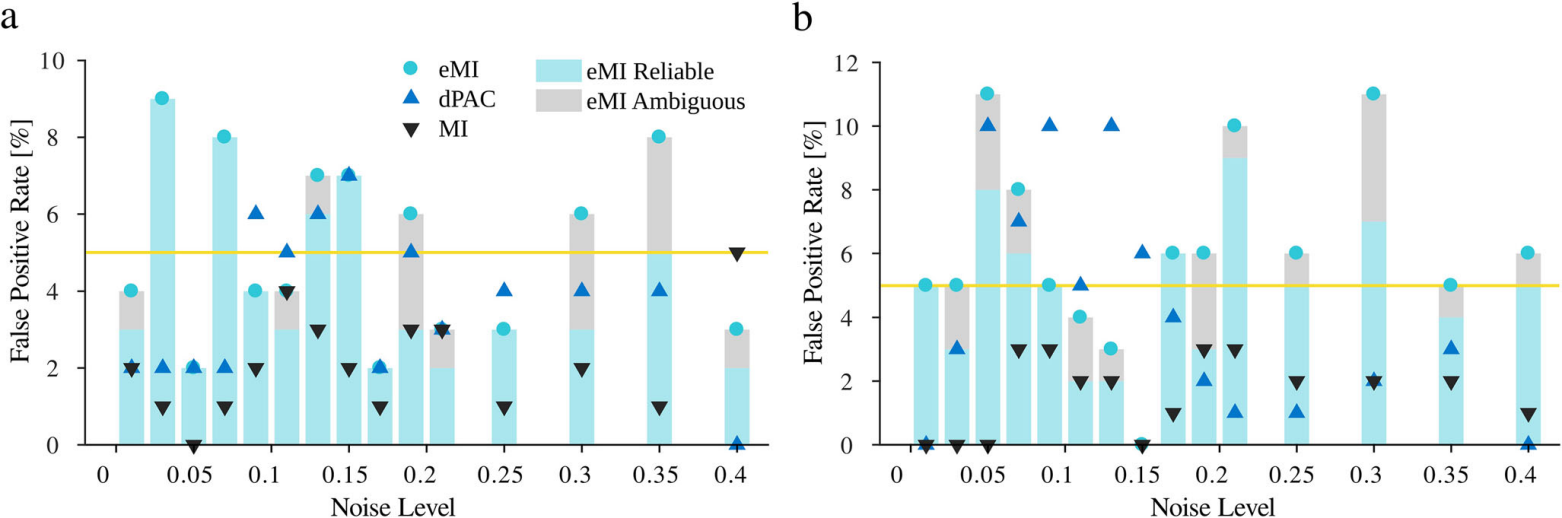
The dependence of False Positive Ratio on *Noise Level* for **a**) random oscillatory bursts (“Random oscillatory bursts”) and **b**) filtered noise (“Filtered noise”.) The yellow line marks the expected percentage of false detections. Turquoise circles—the proposed eMI, black triangles—the reference MI, blue triangles—the reference dPAC. The vertical bars show the proportion of detections marked as reliable (turquoise part) and as ambiguous (gray part) by the eMI algorithm. Δ*f*_*P*_ was set to 1 Hz, and *w* to 5

First, we tested if the methods of surrogate data generation and extreme values statistics control the false-positive detections at the expected level. For this purpose, for each *Noise Level* ranging from 0 to 0.4, we generated 100 realizations of random oscillatory bursts signal (“Random oscillatory bursts”) and 100 realizations of filtered noise signal (“Filtered noise”). Both types of signals by assumption do not contain PAC since the high-frequency amplitude is independent of the low-frequency phase. Hence, we consider as false-positive detection when the comodulogram indicates a statistically significant coupling between any pair of frequencies. The False Positive Ratio is the number of false-positive detections divided by the number of realizations (for each *Noise Level* separately). The obtained results for random oscillatory bursts and filtered noise are shown in Fig. 3a and b, respectively. As expected, the False Positive Ratio for all methods is around $1-\frac {p_{C}}{100}=0.05$ level. The MI method tends to produce False Positive Ratios below the assumed level for both types of generated signals. The eMI and dPAC methods show bigger variance, but they fluctuate as designed around the 5% level.

### The influence of signal properties on PAC detection

Knowing that the False Positive Ratio is controlled at the designed level, we decided to test the influence of signal properties on PAC detection. In the case of the simulated coupled oscillatory bursts (“Coupled oscillatory bursts”), signals with amplitude modulation (“Amplitude modulation”) and multimodal couplings (“Multimodal coupling”), by construction the PAC should occur for the predefined pair of frequencies, i.e., *f*_*P*_ = 6 Hz, and *f*_*A*_ = 77 Hz, therefore we compare the coupling indexes only for this pair of frequencies.

**Fig. 4:**
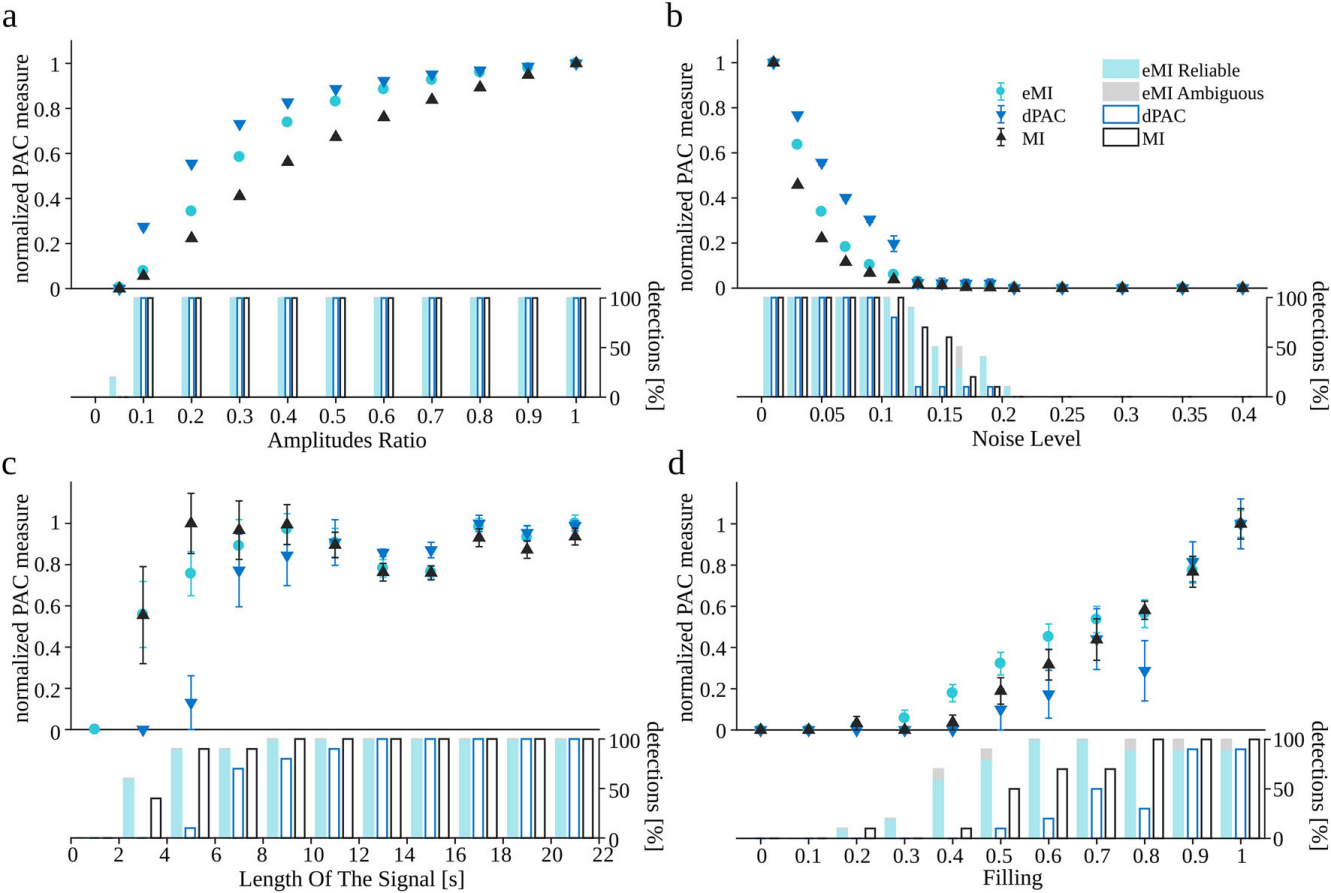
Dependence of coupling measures on the oscillatory bursts signal properties (“Coupled oscillatory bursts”). Turquoise circles eMI, black triangles MI, blue triangles dPAC. All measures were normalized by the maximal value within the investigated range. Marks indicate average across 10 repetitions; error bars indicate SEM. The vertical bars show the percentage of detections. Bars assigned to eMI algorithm additionally depict the proportion of detections marked as reliable (turquoise part) and as ambiguous (gray part). Δ*f*_*P*_ was set to 1 Hz, and *w* to 5, because these parameters yield the most compact coupling region consistent with the simulated coupling between 6 and 77 Hz

We generated ten realizations of the coupled oscillatory bursts signal for each set of parameters, changing only one parameter at a time and setting the others to their default values. The results are shown in Fig. 4. Note that all measures were normalized to their maximal value within the investigated range of the parameter to make the comparison of trends easier.^8^ Besides the relative PAC measure, beneath each plot, there is a barplot indicating the percentage of detected cases. Note that the small value of the relative PAC measure does not preclude a reliable detection of the coupling. A general observation is that all methods exhibit similar characteristics of dependence on the examined parameters.

All the methods manifest a rapid increase of the PAC value with the increase of the *Amplitudes Ratio*, followed by saturation. For the MI method the rate of the changes is the lowest and for dPAC the highest (Fig. 4a). However it is worth noting that all the methods reliably detect the coupling in 100% of cases starting from the *Amplitudes Ratio* 0.1 and eMI detects 20% of cases even for *Amplitudes Ratio* of 0.05.

As the *Noise Level* increases the normalized PAC measures drastically decrease (Fig. 4b). dPAC measure is diminishing the slowest while MI the fastest. All methods fail to detect PAC for *Noise Level* above 0.2. Note, that in the range of *Noise Level* between 0.1 and 0.2 the normalized values of PAC are very small, still the detections are observed and the percentage of detections drops the slowest for eMI.

The normalized PAC measures increase rapidly with the *Length of the signal* (Fig. 4c). MI and eMI reach saturation for signals longer than 5 s, whereas the dPAC stabilizes for signals longer than 7 s. Already a 3 s signal is long enough for MI and eMI to detect coupling, while dPAC needs 5 s at least. Additionally, we observed that eMI produces the smallest variability.

Here, we should note that the dependence of normalized PAC measure on *Length of the signal* is related to the number of averaged cycles of frequency for phase. Increasing the number of cycles increases the signal to noise ratio. As a result, the observed pattern is shifted towards shorter lengths of the signal while increasing the frequency for phase.

The bigger the *Filling* of the signal with coupling, the higher is PAC measure for all methods (Fig. 4d). For eMI and MI the 0.2 *Filling* is enough to detect coupling—it is visible in the detection percentage shown with bars, whereas dPAC needs 0.5 *Filling* at least.

**Fig. 5:**
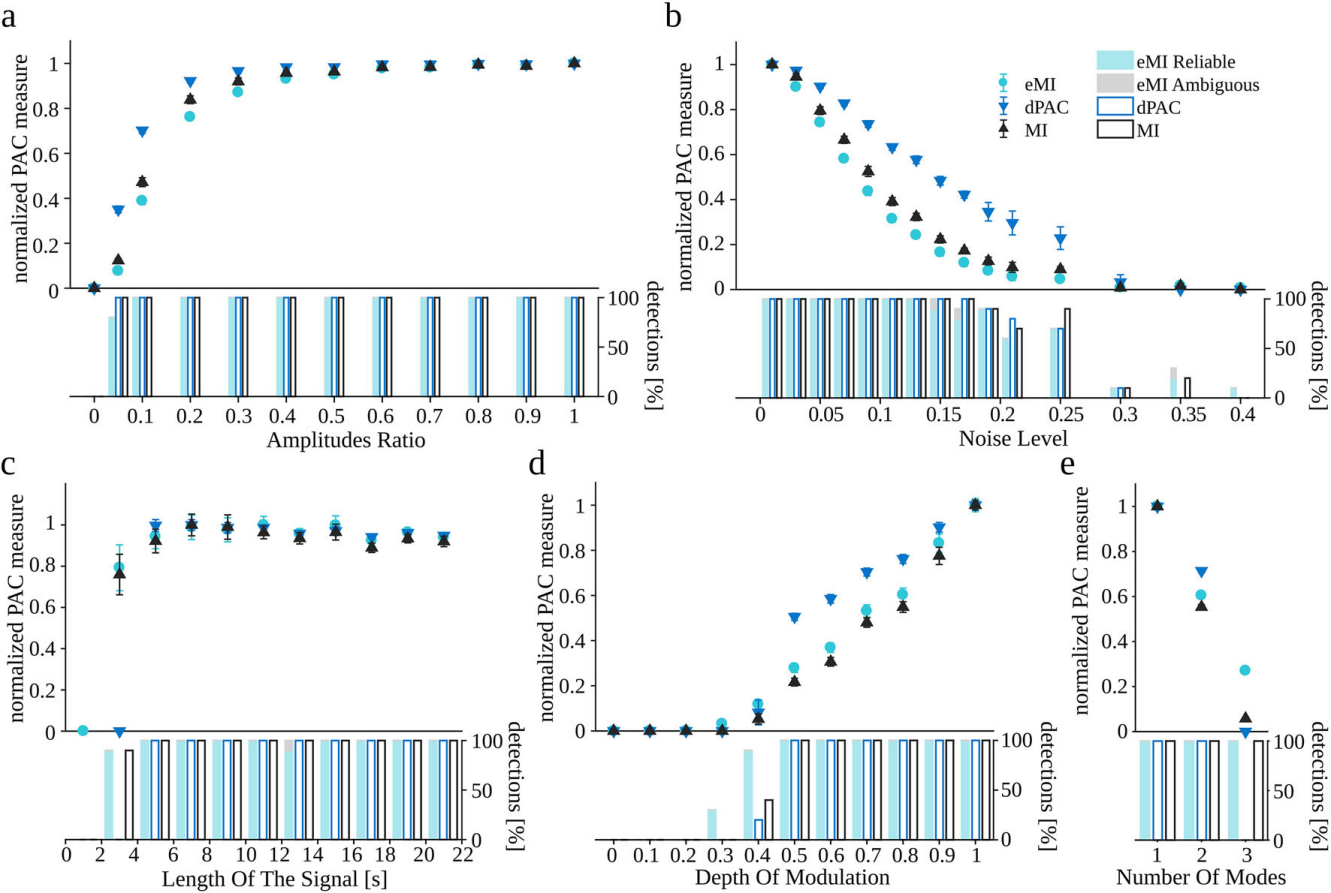
Dependence of coupling measures on (**a–d**) parameters of the signal with amplitude modulation (“Amplitude modulation”) and (e) signal with multimodal coupling (“Multimodal coupling”). Turquoise circles eMI, black triangles MI, blue triangles dPAC. All measures were normalized by the maximal value within the investigated range. Marks indicate average across 10 repetitions; error bars indicate SEM. The vertical bars below each plot show the percentage of detections. Bars assigned to eMI algorithm additionally depict the proportion of detections marked as reliable (turquoise part) and as ambiguous (gray part). Δ*f*_*P*_ was set to 1 Hz, and *w* to 5, because these parameters yield the most compact coupling region consistent with the simulated coupling between 6 and 77 Hz

Further, we wanted to compare the properties of the three methods on models of PAC more commonly assumed in the literature. We generated 10 realizations of the amplitude modulated signal (“Amplitude modulation”) and 10 realizations of the multimodal signal (“Multimodal coupling”) for each set of parameters, changing only one parameter at a time and setting the others to their default values. The results are shown in Fig. 5. The data points on the graphs are the average PAC measure values for the predefined coupled pair of frequencies (*f*_*P*_ = 6 Hz, and *f*_*A*_ = 77 Hz) together with their standard errors. Analogously as in the previous figure, all measures were normalized to the maximal value within the investigated range to make the comparison of the trends easier, and the barplots beneath each plot show the percentage of detected cases.

Here as well, the general observation is that all methods exhibit a similar dependence on the examined parameters. All methods detect PAC if *Amplitudes Ratio* is greater than 0.05 (Fig. 5a). They manifest a rapid increase, followed by saturation of the coupling measure. For eMI the pace of the changes is the lowest and for dPAC the highest.

As the *Noise Level* increases the normalized PAC measures decrease (Fig. 5b). dPAC measure is diminishing the slowest and eMI the fastest. All methods fail to detect most of the PAC cases for *Noise Level* above 0.3 .

The normalized PAC measures increase rapidly with the *Length of the signal* (Fig. 5c). All methods reach saturation for signals longer than 5 s. The 3 s long signal is enough for the MI and eMI to detect coupling, while dPAC needs 5 s at least.

The bigger the *Depth of modulation*, the higher is the PAC measure for all methods (Fig. 5d). For all methods, the *Depth of modulation* = 0.4 is enough to detect coupling. It is worth noting that for eMI, the percentage of detections is the highest at the relatively shallow modulation (0.3–0.4). At deeper modulations, all the methods detect all cases of PAC.

The results for multimodal coupling are presented in Fig. 5e. The consecutive numbers of modes mean that for 1, the coupling is simulated at phase $\frac {4\pi }{5}$, for 2 there is additional high-frequency amplitude augmentation at the $\frac {3\pi }{2}$, and finally, at number 3, there is a further augmentation at around $\frac {\pi }{10}$. In the case of the dPAC method, which relies on mean vector length evaluation, it means more and more cancelation of that vector. Thus for *Number of modes* = 1, all the methods detect all cases of PAC with the maximal value. Also, for *Number of modes* = 2, although the PAC measures are smaller, all the cases are detected by all the methods. For *Number of modes* = 3, dPAC is not seeing the coupling due to the cancellation of the mean vector, the other two methods detect all the cases, although the value of PAC measure is relatively small.

### Identification of a reliable PAC

Data presented in Fig. 4 and 5 compare the PAC measures only at the predefined pairs of frequencies, where the coupling was present by the construction of the signal. Of course, in more realistic settings, it is unknown a priori whether any coupling exists, and, if so, for which pairs of frequencies. In this situation, the whole comodulogram, computed for all the potential pairs of frequencies, needs to be considered.

**Fig. 6:**
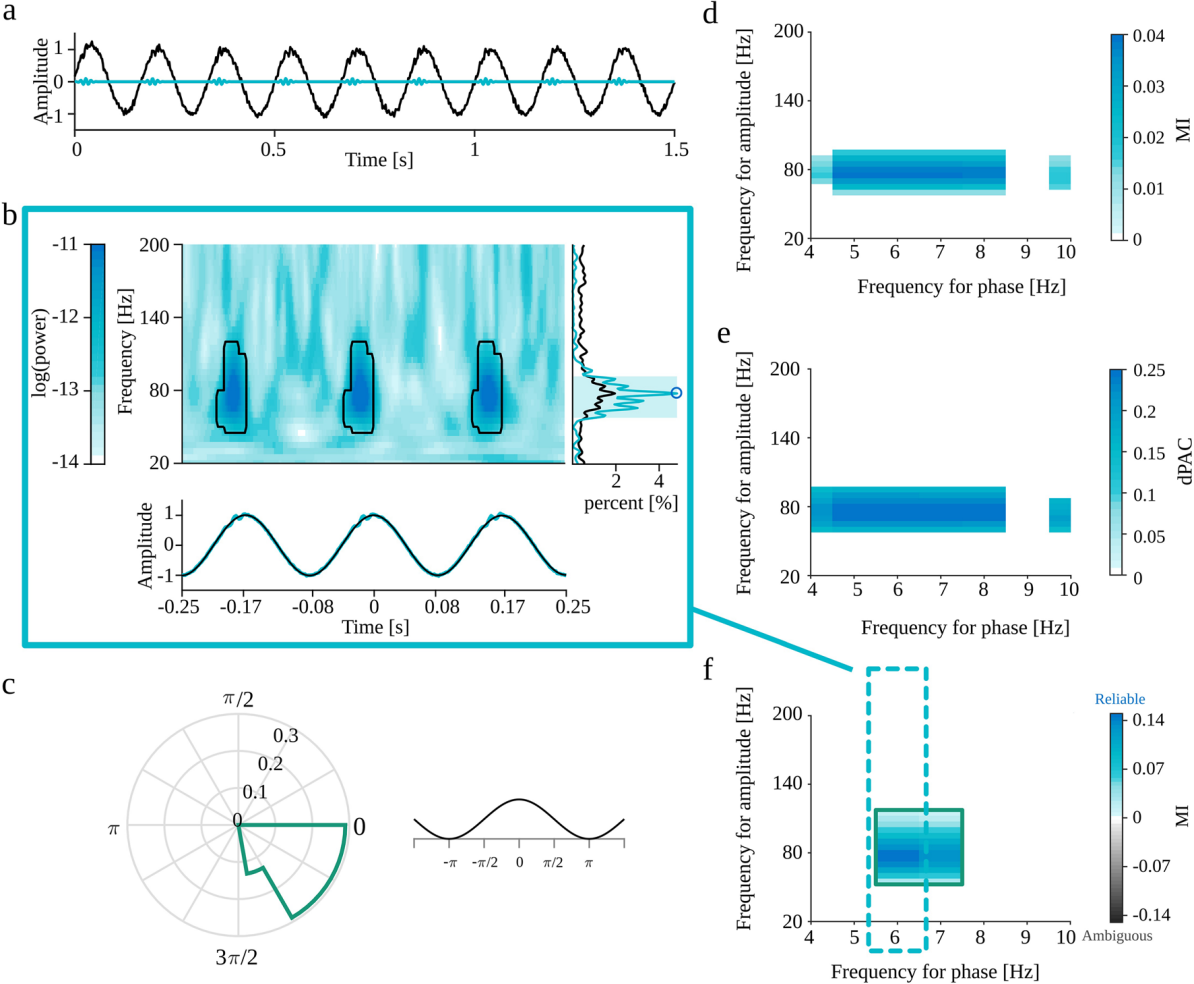
Full results of all the methods for an oscillatory bursts signal (“Coupled oscillatory bursts”) with *Noise Level* set to 0.05 and other parameters set to default values. **a** Black—the synthetic signal, turquoise—the high-frequency component. **b** Auxiliary plots of eMI for *f*_*P*_ = 6 Hz: upper plot—average map $M^{B}_{f_{P}}(t,f)$ with black outline of regions that produce a statistically significant coupling, lower plot—average signal $S^{B}_{f_{P}}(t)$ in turquoise and extracted low-frequency oscillation $SP^{B}_{f_{P}}(t)$ in black, right-side plot—average spectrum $AS_{f_{P}}(f)$ in black, spectrum of the averaged signal $SA_{f_{P}}(f)$ in turquoise, shaded frequency range of maximum in comodulogram $\langle f^{max}_{A}-\frac {1}{2}{\Delta } f^{max}_{A},f^{max}_{A}+\frac {1}{2}{\Delta } f^{max}_{A} \rangle $ and local maximum of spectrum $f^{MAX}_{A}$ marked with a blue circle. **c** Polar phase histogram (eMI) for the outlined region on the comodulogram. A fragment of a cosine function at the right visualizes the relation between the phase and the time-course of the low-frequency component. **d** Comodulogram obtained with MI. **e** Comodulogram obtained with dPAC. **f** Comodulogram obtained with eMI presenting the strength of significant coupling, and it’s assessment as *Reliable* or *Ambiguous* encoded in colors. Δ*f*_*P*_ was set to 1 Hz, and *w* to 5, because these parameters yield the most compact coupling region consistent with the simulated coupling between 6 and 77 Hz

Figure 6d, e and f show an example evaluation of the full comodulogram by MI, dPAC and eMI techniques, respectively, for the oscillatory bursts signal (displayed in Fig. 6a) generated as described in “Coupled oscillatory bursts” with *Noise Level* set to 0.05 and other parameters set to default values (coupling between 6 and 77 Hz). We observe that eMI is considerably more specific than both the reference methods. The eMI comodulogram shows maximum coupling at the predefined frequency for phase *f*_*P*_ = 6 Hz in contrast to the reference methods, where almost the whole low-frequency band displays strong coupling. The specificity in frequency for amplitude is comparable for all methods for this example. However, the properties of the wavelet transform used in eMI impose the trade-off between the frequency and time resolution. The higher the frequency, the worse is the frequency resolution, and better is time resolution. Those properties of eMI are in contrast to constant frequency resolution of MI and dPAC methods resulting from the constant bandwidth of the applied filters.

The auxiliary eMI results, as well as polar phase-histogram, are presented in Fig. 6b and c respectively. They provide additional information about the properties of the coupling. We can see that the bursts of high-frequency oscillations are quite narrow, limited in frequency dimension and that they occur just before the peaks of the low-frequency cycle. In this case of highly synchronized synthetic coupling, even the averaged signal in Fig. 6b reveals bursts of high-frequency oscillation. It is reflected in the averaged signal’s spectrum in the form of a prominent peak, which is congruent with the maximum in comodulogram. This concurrence ensures the assignment of a *Reliable* coupling label.

### Finding signatures of spurious PAC with the eMI auxiliary plots

In this subsection, we focus on how the auxiliary plots generated by the eMI toolbox can help in getting a more in-depth insight into the nature of the coupling, what are the signatures of epiphenomenal origins of the couplings and how the automatic label assignment works.

**Fig. 7:**
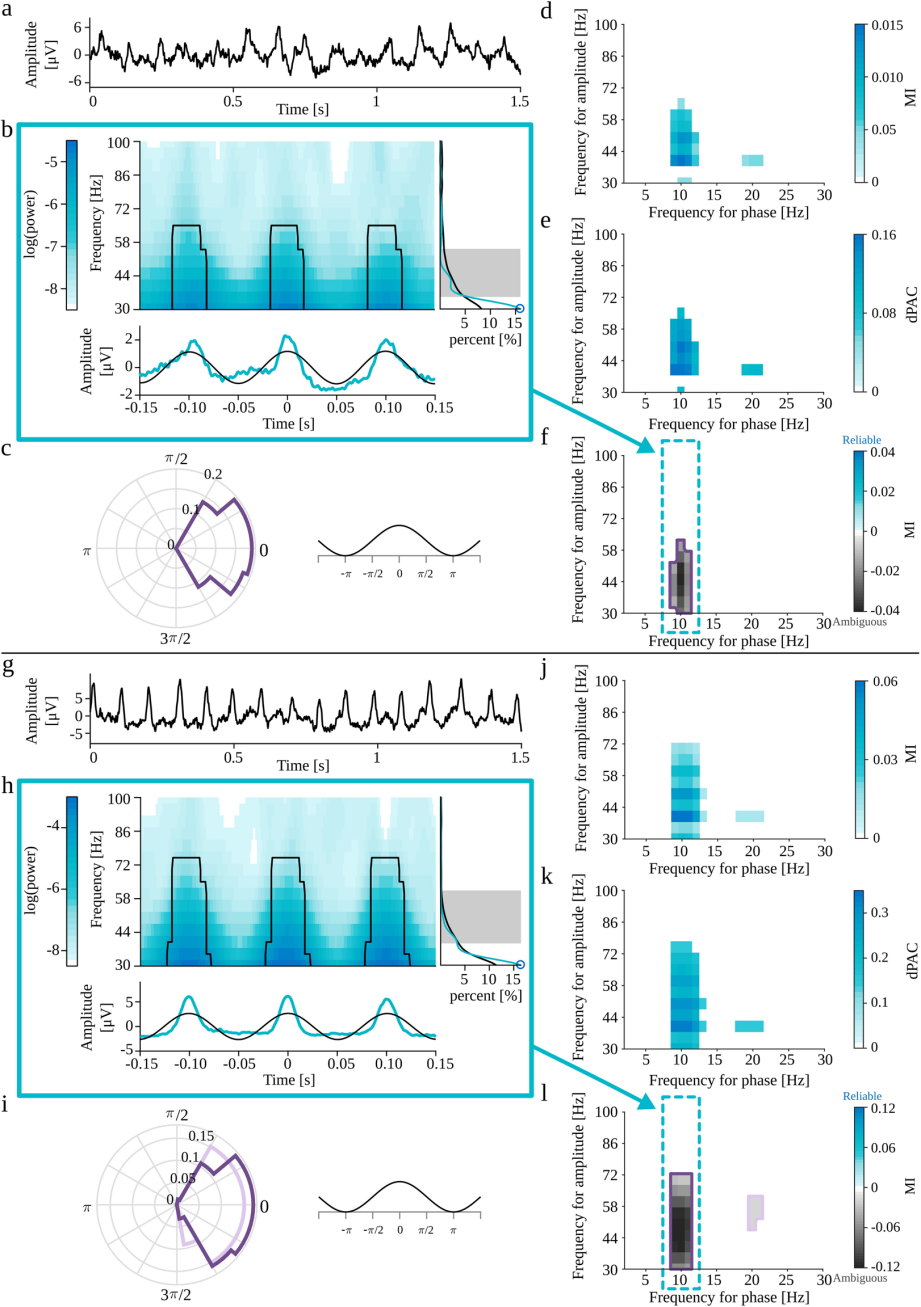
Full results of PAC detection for periodic trains of Gaussian functions (“Periodic trains of Gaussian functions”) with Gaussian spike amplitude set to 2 (**a-f**) and 5 (**g-l**) standard deviations of the background EEG signal. **a,g**) analyzed signal. b,h) Auxiliary plots of eMI for *f*_*P*_ = 10 Hz, corresponding to the region of comodulogram indicated by the turquoise arrow and frame in panels f,l): upper plot—average map $M^{B}_{f_{P}}(t,f)$ with black outline of regions that produce a statistically significant coupling, lower plot—average signal $S^{B}_{f_{P}}(t)$ in turquoise and low-frequency oscillation $SP^{B}_{f_{P}}(t)$ in black, right-side plot—average spectrum $AS_{f_{P}}(f)$ in black, spectrum of averaged signal $SA_{f_{P}}(f)$ in turquoise, shaded frequency range of maximum in comodulogram, and local maximum of the spectrum marked with a blue circle. **c,i**) Polar phase histogram (eMI) for each outlined region from the comodulograms **f,l**) **d,j**) Comodulogram for MI. **e,k**) Comodulogram for dPAC. **f,l**) Comodulogram for eMI presenting strength of significant coupling and it’s assignment as *Reliable* or *Ambiguous*. Each separate region is outlined with a different color, which acts as a legend for a polar phase histogram. Δ*f*_*P*_ was set to 2 Hz and *w* to 5

Most of the spurious PAC detections originate from the non-sinusoidal shape of the analyzed oscillatory signal. Such waveforms can be expressed by Fourier transform as a series of sinusoids. In some cases, the power of higher harmonics is sufficient to produce a false detection of coupling between the base frequency and higher harmonics. Gerber et al. (2016) noted that a semi-periodic occurrence of sharp waveform could yield spurious phase-amplitude coupling. To test this, we generated the periodic trains of Gaussian functions (“Periodic trains of Gaussian functions”). The Fourier power spectrum of a Gaussian function with width *σ*_*t*_ is also a Gaussian function with width $\sigma _{w}=\frac {1}{\sigma _{t}}$. However, the superposition of those functions in a periodic series produces spectral harmonic structure, with a base frequency equal to the inverse of the interval between the Gaussian spikes, with a fall-off consistent with a spectrum of an individual spike. Figure 7a-f present the results for the variant with Gaussian spike amplitude set to 2 standard deviations of the background EEG signal, whereas Fig. 7g-l shows the results for the Gaussian spike amplitude set to 5 standard deviations. We expect spurious coupling between the average frequency of occurrence of Gaussian spikes (i.e., 10 Hz) and the wide range of high-frequencies composing those structures in the frequency domain. This coupling, for both signals, is detected by all methods (Fig. 7d-f, j-l). Most of the comodulograms also display the coupling with the second harmonic (Fig. 7d, e, j, k, l). This property may suggest the epiphenomenal origins of coupling. The auxiliary plots from the eMI method presented in Fig. 7b, h, as well as polar phase-histograms (Fig. 7c, i) provide additional information about the characteristics of the coupling. We can observe that coupling occurs where the average signal undergoes abrupt changes in amplitude. Also, we can note that the averaged signal does not contain a sinusoidal low-frequency component as extracted through filtration, and the time-frequency maps display a wide range of coupled frequencies. The average spectrum does not contain any prominent peaks, whereas the spectrum of the averaged signal displays characteristic harmonic structure with a maximum not congruent with the maximum in comodulogram. All of those indicators of epiphenomenal origins are much more pronounced for the signal with higher Gaussian spikes amplitude. Visual inspection of auxiliary plots allows us to integrate all of that additional information and confirm the automatically assigned *Ambiguous* origins of the coupling.

**Fig. 8:**
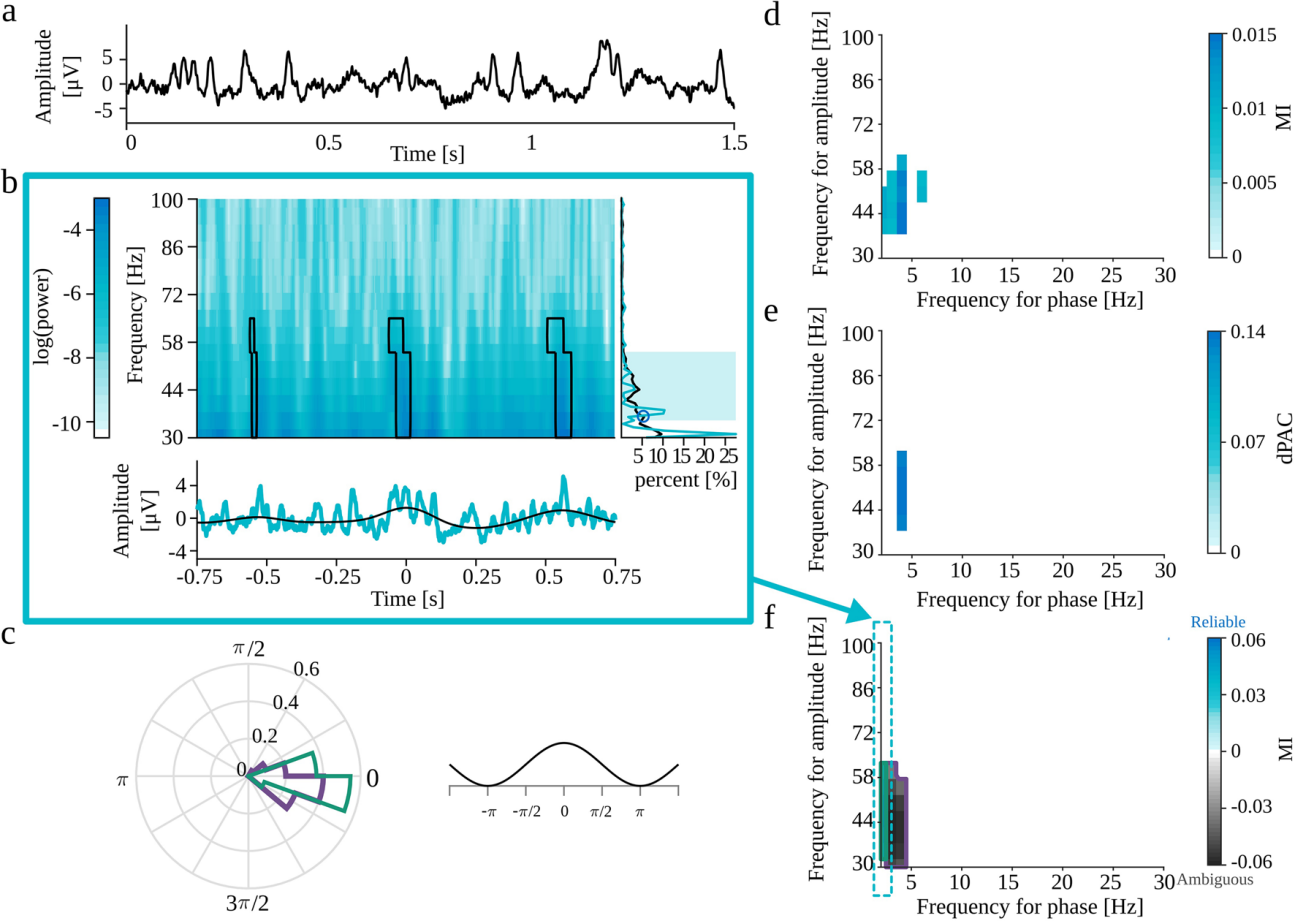
Full results of PAC detection for non-periodic trains of Gaussian functions (“Non-periodic trains of Gaussian functions”) with Gaussian spike amplitude set to and 5 standard deviations of the background EEG signal. **a** analyzed signal. **b** Auxiliary plots of eMI for *f*_*P*_ = 2 Hz, corresponding to the region of comodulogram indicated by the turquoise arrow and frame in panel f): upper plot—average map $M^{B}_{f_{P}}(t,f)$ with black outline of regions that produce a statistically significant coupling, lower plot—average signal $S^{B}_{f_{P}}(t)$ in turquoise and extracted low-frequency oscillation $SP^{B}_{f_{P}}(t)$ in black, right-side plot—average spectrum $AS_{f_{P}}(f)$ in black, spectrum of averaged signal $SA_{f_{P}}(f)$ in turquoise, shaded frequency range of maximum in comodulogram, and local spectrum maximum marked with a blue circle. **c** Polar phase histogram (eMI) for each outlined region from the comodulogram. **d** Comodulogram for MI. **e** Comodulogram for dPAC. f) Comodulogram for eMI presenting strength of significant coupling and it’s assignment as *Reliable* or *Ambiguous*. Each separate region is outlined with a different color, which acts as a legend for a polar phase histogram. Δ*f*_*P*_ was set to 2 Hz and *w* to 5

Gerber et al. (2016) also claims that the strict periodicity is not necessary for PAC as long as the sharp transients are spaced sufficiently apart for a given frequency for phase such that the peaks of this slow frequency can align with the sharp waves. In order to investigate this notion, we generated the non-periodic trains of Gaussian functions (“Non-periodic trains of Gaussian functions”). We expect that due to the finite length of the signal, the random localization of sharp transients will produce a non-uniform distribution of the intervals. In some realizations, this effect of heterogeneity may lead to the detection of PAC between this random preferential frequency of occurrence of Gaussian functions and a wide range of high-frequency sinusoids composing those structures in the Fourier domain. The signal with Gaussian spike amplitude set to 2 standard deviations of the background EEG signal did not produce statistically significant PAC. Fig. 8a–f shows the results for the Gaussian spike amplitude set to 5. All methods detect postulated coupling between frequency bands ranging from 2 to 5 Hz in frequency for phase, and from 30 to 60 Hz in frequency for amplitude (Fig. 8d–f). Just by looking at MI and dPAC comodulograms, we do not have any indications that this is a spurious coupling. Most of the labels automatically assigned by eMI correctly suggest ambiguous origins of this coupling. However, there is one region with falsely assigned *Reliable* label, which should be a reminder that eMI also has some limitations. The automatic label assignment sometimes may be wrong, which is why we strongly suggest double-check it by visual inspection of auxiliary plots (Fig. 8b and c). In this case, we can observe that the coupling occurs where the average signal undergoes abrupt changes in amplitude. Also, we can note that the averaged signal does not contain a sinusoidal low-frequency component as extracted through filtration, and the time-frequency maps display a wide range of coupled frequencies. The average spectrum does not contain any prominent peaks, whereas the spectrum of the averaged signal display characteristic harmonic structure. All of those features indicate the epiphenomenal origins of the coupling. As part of a thorough inspection of this case, we tried to reproduce this effect, and the conclusion is that this is not a stable result. In some realizations of the random locations of the Gaussian spikes, there is no detected PAC at all. Importantly, the comodulograms from all three methods were consistent for each realization. These additional considerations confirm that the coupling detected in the model of non-periodic trains of Gaussian is an epiphenomenon due to random process on finite time interval.

### In vivo data

So far, PAC was analyzed in different simulated settings. We showed that all described methods detect expected coupling. Nonetheless, while using MI and dPAC it was impossible to infer coupling origins. Whereas eMI provided automatically and mostly correctly assigned *Reliable* or *Ambiguous* labels and auxiliary plots which allowed to get additional information on the characteristic of the coupling and to double-check the assigned labels. In this section, we wanted to investigate the performance of all methods in vivo settings.

**Fig. 9:**
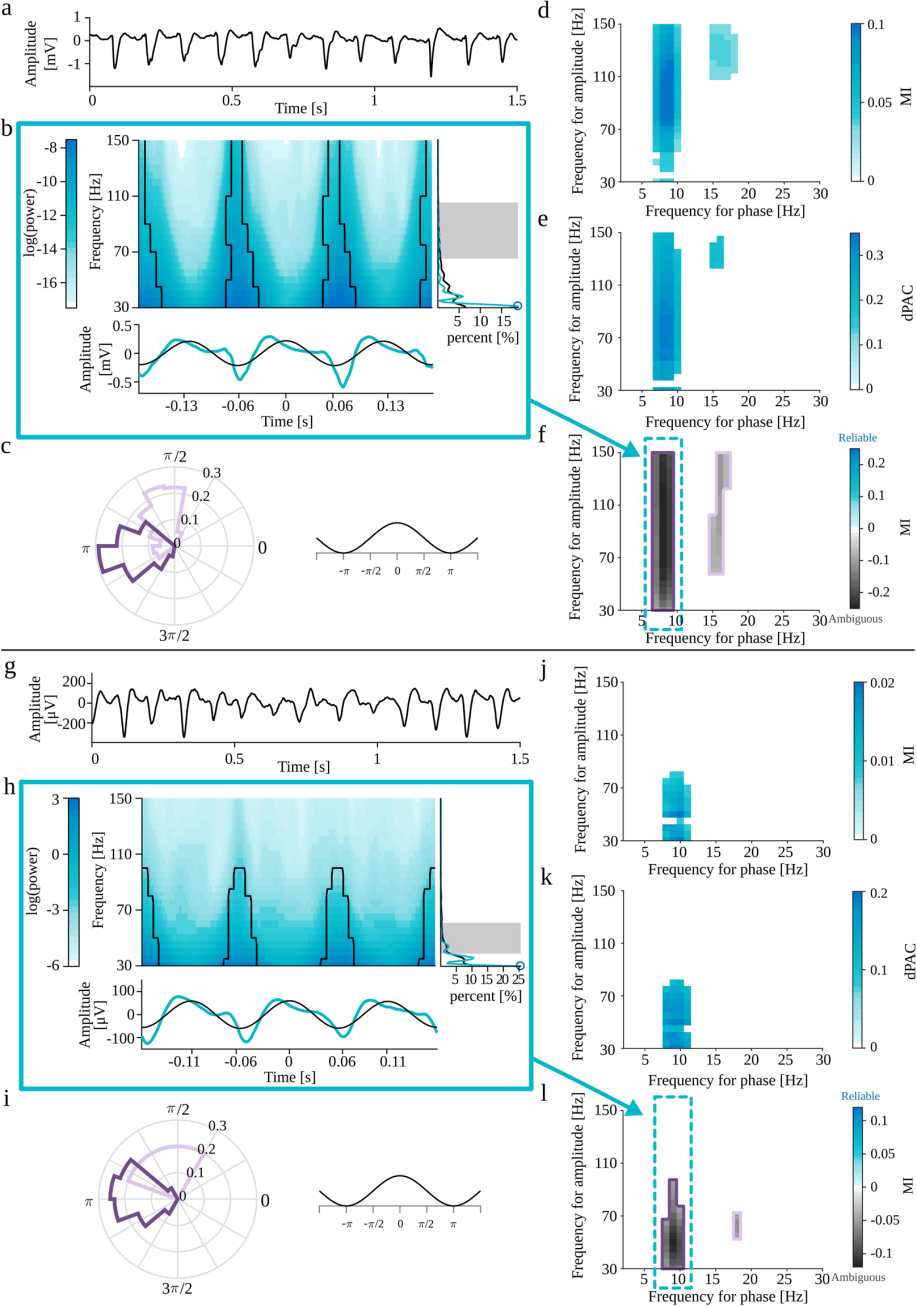
Full results of PAC detection for (**a-f**) recording from rat with epilepsy (“Recording from a rat with epilepsy” and (**g-l**) for mu rhythm in ECoG signal (“Mu rhythm in ECoG signal”). a,g) Black—the analyzed signal. b,h) Auxiliary plots of eMI for *f*_*P*_ = 8 Hz (**b**) or *f*_*P*_ = 9 Hz (**h**), corresponding to the region of comodulogram indicated by the turquoise arrow and frame in panel f,l): upper plot—average map $M^{B}_{f_{P}}(t,f)$ with black outline of regions that produce a statistically significant coupling, lower plot—average signal $S^{B}_{f_{P}}(t)$ in turquoise and extracted low-frequency oscillation $SP^{B}_{f_{P}}(t)$ in black, right-side plot—average spectrum $AS_{f_{P}}(f)$ in black, spectrum of averaged signal $SA_{f_{P}}(f)$ in turquoise, shaded frequency range of maximum in comodulogram and local spectrum maximum marked with a blue circle. **c,i**) Polar phase histogram (eMI) for each outlined region from the comodulograms f,l) d,j) Comodulogram for MI. **e,k**) Comodulogram for dPAC. f,l) Comodulogram for eMI presenting strength of significant coupling and it’s assignment as *Reliable* or *Ambiguous*. Each separate region is outlined with a different color, which acts as a legend for a polar phase histogram. Δ*f*_*P*_ was set to 2 Hz and *w* to 5

Figure 9a shows the recording from a rat with epilepsy (“Recording from a rat with epilepsy”)—an example of a real signal containing presumably false PAC. In this case, we may expect a wave-dependent coupling between the frequency of occurrence of sharp transients ($\sim $8 Hz) and the wide range of high-frequencies composing those structures in the frequency domain. Indeed, this coupling is detected by all methods (Fig. 9d, e and f). Looking at the MI and dPAC comodulograms, the only indication of a waveform-dependent origin could be the presence of the second harmonic. While labels in eMI comodulogram inform us about the ambiguous origins of the coupling. Importantly, the auxiliary plots from the eMI method presented in Fig. 9b and polar phase-plot (Fig. 9c) provide additional information about the characteristics of the coupling. We can observe that coupling occurs where the average signal contains sharp transients. The time-frequency maps display a wide range of coupled frequencies, and both average spectrum and spectrum of average signal manifest harmonic structure. Combining all of those features confirm the ambiguous origins of the coupling.

Figure 9g shows the mu rhythm in ECoG signal (“Mu rhythm in ECoG signal”)—the second example of a real signal containing presumably spurious PAC. In this case, we may also expect a wave-dependent coupling between the frequency of occurrence of sharp transients ($\sim $9 Hz) and the wide range of high-frequencies composing those structures in the frequency domain. As predicted, all methods detect coupling (Fig. 9j, k and l) which eMI additionally assessed as ambiguous. MI and dPAC comodulograms contain no indication of a waveform-dependent origins. Additionally, the auxiliary plots (Fig. 9h and i) provide information about the characteristics of the coupling. We can observe that coupling occurs where the average signal contains sharp transients. The time-frequency maps display a wide range of coupled frequencies and spectrum of average signal exhibit weak harmonic structure. All of those observations suggest properly assigned ambiguous origins of the coupling.

**Fig. 10:**
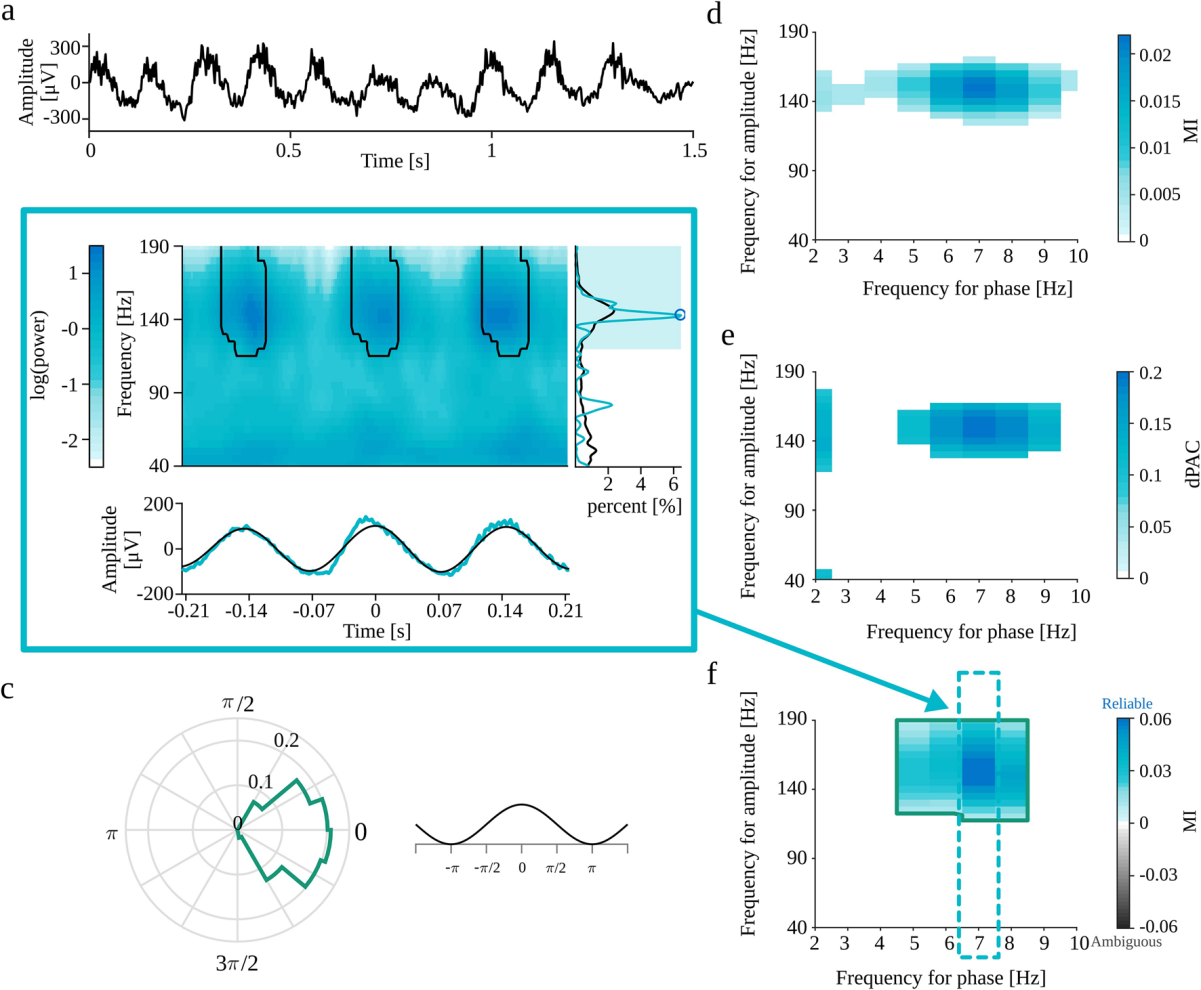
Full results of all methods for a 20 seconds fragment of recording from rat after ketamine injection (“Recordings from rats after ketamine injection”), 6 minutes after injection of 25 mg/kg ketamine. **a** Example section of the analyzed signal. **b** Auxiliary plots of eMI for *f*_*P*_ = 7 Hz: upper plot—average map $M^{B}_{f_{P}}(t,f)$ with black outline of regions that produce a statistically significant coupling, lower plot—average signal $S^{B}_{f_{P}}(t)$ in turquoise and extracted low-frequency oscillation $SP^{B}_{f_{P}}(t)$ in black, right-side plot—average spectrum $AS_{f_{P}}(f)$ in black, spectrum of averaged signal $SA_{f_{P}}(f)$ in turquoise, shaded frequency range of maximum in comodulogram, and local maximum of the spectrum marked with a blue circle. c) Polar phase histogram (eMI) for the outlined region from the comodulogram d) Comodulogram for MI. e) Comodulogram for dPAC. f) Comodulogram for eMI presenting strength of significant coupling and it’s assignment as *Reliable*. The area outlined with green color corresponds to the polar phase histogram. Δ*f*_*P*_ was set to 2 Hz and *w* to 7

The recordings from rats after ketamine injection (“Recordings from rats after ketamine injection”) were selected as an opportunity to examine signals with assumed physiological PAC origins. In this case, we expect ketamine to induce High-Frequency Oscillation (HFO), around 150 Hz, which couple with the theta frequency range. Those long recordings were analyzed as a sequence of 20 s epochs for each rat (n = 7). The results for a selected rat for a 20 s section occurring 6 minutes after injection of 25 mg/kg ketamine are presented in Fig.10a–f. As shown in Fig.10d–f, all methods detect coupling between an HFO around 150 Hz, and low-frequency oscillation from theta range, centered around 7 Hz. As predicted, the eMI comodulogram inform us about the assigned *Reliable* label. Auxiliary plots presented in Fig.10b and c provide additional information. They demonstrate that the bursts of high-frequency oscillations occupy a limited frequency range and appear around the peak of a 7 Hz oscillation. Also, we can note that the averaged signal does contain a sinusoidal low-frequency component as extracted through filtration, and it does not contain abrupt amplitude changes. Both the average spectrum and spectrum of averaged signal contain a prominent peak in the coupled region. Together, these findings strongly suggest the physiological origins of the coupling.

To summarize the information from a batch of comodulograms, we present their time evolution in a collated form. For each time point and each frequency for amplitude, we selected the maximal value of PAC measure (across all frequencies for phase) labeled as *Reliable*. Presented results are controlled with *False Discovery Rate* procedure at the level of *q* = 0.05.

**Fig. 11:**
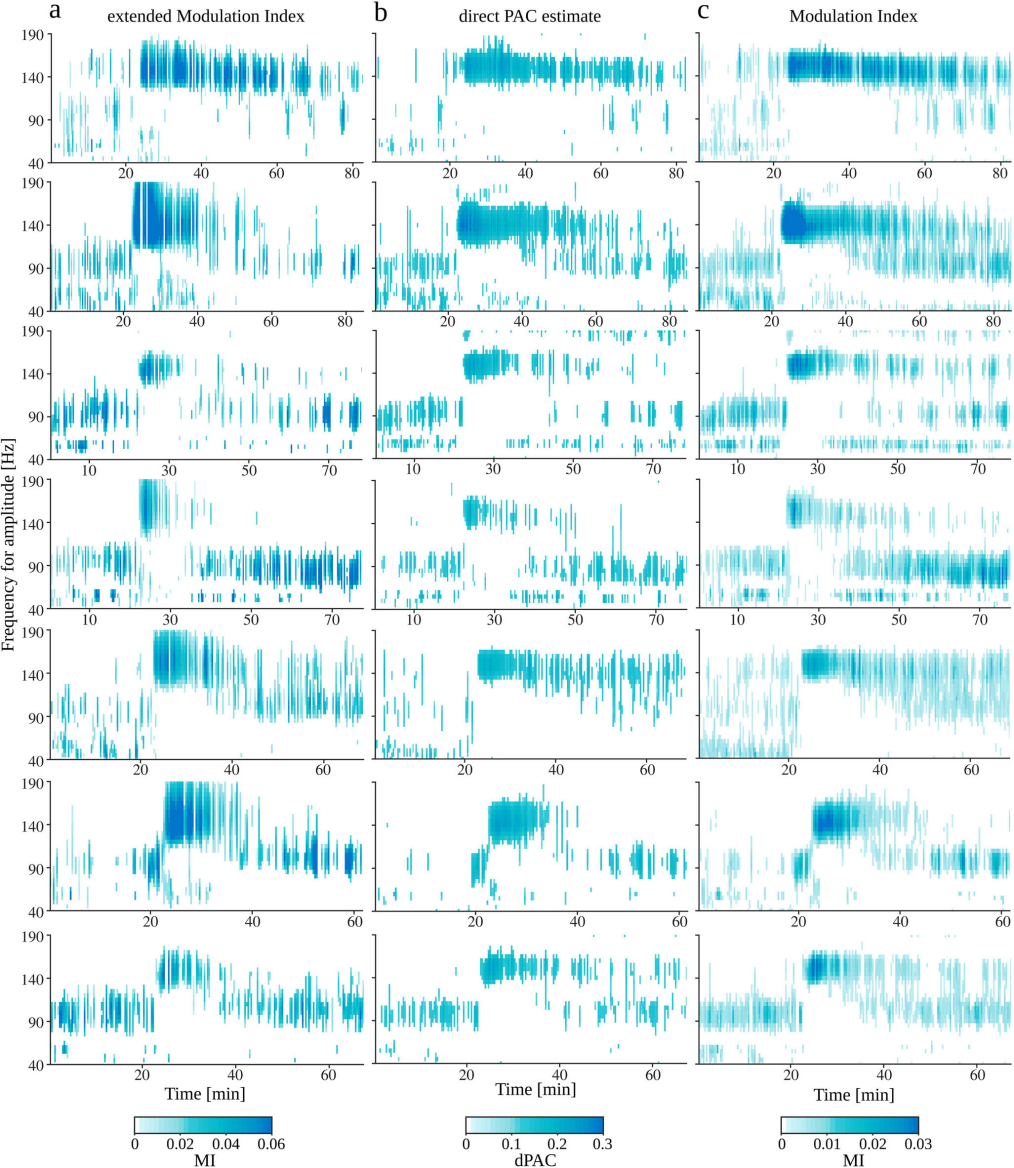
Full results of PAC detection for recordings from rats after ketamine injection (“Recordings from rats after ketamine injection”). Results are presented for each examined rat (n = 7, in consecutive rows) and for all methods (in consecutive columns): **a** eMI (here we present only coupling labeled as *Reliable*), **b** dPAC, **c** MI. Ketamine was injected around 21^*st*^ minute of recording. The results are corrected with *False Discovery Rate* with *q* = 0.05

In this collated form we examined the whole recording session for all seven rats and for all three methods (Fig.11 columns a, b, c for methods eMI, dPAC, MI respectively). 25 mg/kg ketamine was injected systemically after 21 minutes of recording. The coupling with an HFO (around 150 Hz) appears shortly after ketamine injection and lasts for up to 60 minutes. We can also observe the coupling with high-gamma (around 90 Hz), which is visible before injection, substantially weaker for the first 20 minutes after injection, and begins to normalize after that.

## Discussion

### Comparison of eMI and the reference methods

The main goal of this study was to develop, test, and compare a novel eMI method with existing established methods of PAC detection. To ensure reliable comparison, we tested whether the methods of surrogate data generation combined with extreme values statistics produce the expected percentage of False Positives (“False Positive Ratio”). As intended, the proposed statistical approach controlled the False Positive Ratio at the predefined level around 5% for all the tested methods. The spread of results around the assumed 5% is due to the relatively low number of repetitions. The obtained result ensures the correct conclusion about the significance of the effects presented on the comodulograms for all methods.

This conclusion allowed us to compare the other results reliably. Our main observation regarding the influence of signal properties on PAC detection (“The influence of signal properties on PAC detection”) is that all the methods exhibit similar characteristics of dependence on the examined parameters. There are small differences in the rise or fall rate, but essentially their performance in terms of sensitivity is comparable (with occasionally slightly higher detection rate for eMI than for reference methods). All methods detect coupling when the *Amplitudes Ratio* is above 0.1–0.05 and *Noise Level* below 0.2–0.3 for coupled oscillatory bursts and amplitude modulation model, respectively. The differences in limiting values of *Length of the signal* may result from the dPAC strategy to avoid edge effects of filtration, by removing the first and last second of signal from the analysis. Hence, a 3 s signal is enough for MI and eMI to detect coupling, while dPAC needs 5 s at least. For eMI and MI a *Filling* of 0.2 is enough to detect coupling, whereas dPAC needs *Filling* of at least 0.5. For all the methods, the *Depth of modulation* equal to 0.4 is enough to detect coupling. The only strongly differentiating property is that dPAC is incapable of detecting multimodal coupling when the coupled phases presented as vectors in polar coordinates cancel each other out.

The most important difference between eMI and the reference methods is noticeable while making an attempt to infer the coupling origins (“Identification of a reliable PAC” and “Finding signatures of spurious PAC with the eMI auxiliary plots”). The only result given by MI and dPAC is the comodulogram. Hence, the only indication of false coupling is the presence of harmonics. In contrast, eMI provides not only an automatic origins label assignment but also a compact form of auxiliary plots, which support the interpretation and further investigation of observed coupling. Comparison of Figs. 6 and 7 and 8 exemplifies this observation. The proposed polar phase-histograms could be useful when studying the phase relation of couplings observed for different pairs of frequencies or obtained for different experimental conditions.

There is an additional difference between eMI and the reference methods. Analysis of artificial coupled oscillatory bursts (Fig. 6) revealed eMI has higher specificity in determining the frequency for phase. This feature is visible also in results of analysis of less artificial (Figs. 7 and 8) and real data (Figs. 9 and 10) but in more subtle way. This property is partially explainable by the type of filters used to extract the low-frequency oscillation, and partially because of the focus of the analysis on the meaningful low-frequencies. The specificity in frequency for amplitude of eMI is dependent on the frequency. It follows the properties of wavelet transform. This feature of eMI is in contrast to constant frequency for amplitude resolution of MI and dPAC methods.

### Recording from rat with epilepsy

Phase-amplitude coupling is reported widely in electrographic seizures recordings (Samiee et al. 2018; Guirgis et al. 2015). The epileptic data used in this study also exhibited statistically significant PAC (Fig. 9a–f). One of the characteristic features of epileptic seizures, observed in the reported case, is the presence of periodic spiky transient structures, which are postulated to be generated by a nonlinear dynamic process (Pijn et al. 1997). Based on the visual inspection of examined recording, which contains a series of characteristic waveforms described above and no trace of separate high-frequency oscillation, we hypothesized a waveform-dependent, thus epiphenomenal origin of the detected coupling. Consistent with those assumptions eMI labeled PAC as *Ambiguous* which is confirmed by visual analysis of auxiliary plots.

### Mu rhythm in ECoG signal

Mu rhythm is an oscillation characteristic for sensory-motor areas. Its frequency range overlaps with the alpha range (8–12 Hz). Besides localization, the mu and alpha rhythms differ in shape—mu waves are typically characterized by periodic sharp transients in the shape of the Greek letter *μ*. This signal has been previously tested for the presence of phase-amplitude coupling by Gerber et al. (2016). The authors found statistically significant coupling between 10 Hz, and a broad range of gamma frequencies. Concerning the specific shape of mu waves, they postulated epiphenomenal origins of the coupling.

In this study, the examined fragment of mu oscillations also proved to contain significant PAC (Fig. 9g–l). In line with expectations, the eMI algorithm suggested *Ambiguous* origins. Visual inspection of auxiliary plots supports this conclusion.

### Recordings from rats after ketamine injection

Ketamine, a psychoactive compound, has been widely reported to induce abnormal HFO in freely moving rodents (Hunt et al. 2006; Cordon et al. 2015; Caixeta et al. 2013; Pittman-Polletta et al. 2018). Recent work suggests the OB is an important generator of this activity, which can impose this activity in ventral striatal areas (Hunt et al. 2019). In the OB, it is well-established that theta can couple to gamma oscillations (40-80 Hz) (for review see (Kay 2014)). Here, we demonstrate that, in the rodent OB, theta also couples to frequencies above the classical gamma band, namely HFO. This finding is consistent with reports from other brain regions showing theta/HFO CFC in striatal and cortical areas (Cordon et al. 2015; Pittman-Polletta et al. 2018; Caixeta et al. 2013; Ye et al. 2018).

The eMI methodology, proposed here, demonstrated moderate to strong theta/HFO coupling post ketamine in all seven rats. Depth profile studies across the OB have shown that ketamine-HFO dramatically changes in power and reverses phase close to the mitral layer (Hunt et al. 2019). For this reason, the precise recording site within the OB may largely influence the degree of coupling observed, with greater coupling proportional to HFO power. Although all electrodes were located with the OB, for technical reasons, the exact laminar location was not determined. Further studies are warranted to determine more precisely how theta/HFO coupling may be related to specific layers within the OB.

Notably, the time-course of theta/HFO coupling (immediately after injection of 25 mg/kg ketamine, pronounced for about 15 min. and lasting up to 60 min.) was very similar to the reported time-course of behavioural activation at the same dose (Hunt et al. 2006). This is consistent with Cordon et al. (2015), who reported that theta/HFO coupling was a key signature of ketamine-induced hyperlocomotion for cortico-basal ganglia structures.

### Interpretation of PAC

The works of Kramer et al. (2008) and Aru et al. (2015) and Gerber et al. (2016) have drawn attention to the challenges of meaningful interpretation of CFC results reported in the literature. Since the aim of this study was to propose a method that supports resolving the problem of interpreting PAC, here we review the list of recommendations presented in Aru et al. (2015) and discuss the extent to which the eMI toolbox supports them.

Presence of oscillations: A precondition for PAC is the presence of a slow oscillation and a high-frequency oscillation modulated by this lower frequency. In eMI toolbox only significant and meaningful low-frequency oscillations are subjected to further analysis (procedure described in “Obtaining significant low-frequency oscillation”). Whereas, the requirement of presence of high-frequency oscillation is built in the heuristics that the statistically significant coupling is reliable when the local maximum in the spectrum within the frequency for amplitude and local maximum in the comodulogram is congruent (“Assignment of Reliable/Ambiguous label”).
Selection of bandwidths: The choice of the frequency band for testing the power of modulation in high frequencies is crucial for observing these modulations. The natural choice here are methods of estimation of the power spectral density in the time-frequency domain.
  We, like Nakhnikian et al. (2016) and Caiola et al. (2019), used a method based on continuous wavelet transform. We released the constraint of the logarithmically spaced frequency vector utilized in Nakhnikian et al. (2016). It makes the eMI method more adaptive to the signal features. We have shown that MI measure essentially does not depend on the selected wavelet wave number *w* (in reasonable range) and bandwidth of the low-frequency filter Δ*f*_*P*_ (Fig. 2).
Interpretation of instantaneous phase: Any potential disturbances in phase continuity should appear as disturbances in the smooth time course of the average low-frequency signal $SP^{B}_{f_{P}}(t)$. The relation between modulated high-frequency power and modulating low-frequency phase can be identified by comparing the $M^{B}_{f_{P}}(t,f)$ map with the course of the average low-frequency signal $SP^{B}_{f_{P}}(t)$.
Precision: In the case of the eMI method, the potential source of inaccuracies in the estimation of energy density distributions arise from edge effects. We minimize this problem by cutting off fragments distorted by edge effects. That is why we recommend selecting sections of signal *s*(*t*) longer than the epoch of interest.
Testing for non-linearities: Aru et al. (2015) recommends testing the contribution of non-linearity and phase-phase coupling to the observed relationships between frequencies. As noted by Gerber et al. (2016): “Harmonics in the frequency spectrum and in the comodulogram may be an indication that the raw signal contains non-sinusoidal periodic waveforms that may contain higher-frequency components [...]. A wide non-harmonic range of frequencies for phase may indicate a non-periodic sequence of sharp waves.” Because of that, we decided to label the whole harmonic structure, with ambiguous base frequency, as *Ambiguous* (“Assignment of Reliable/Ambiguous label”). Additionally, we suggest qualitative observation of maps $M^{B}_{f_{P}}(t,f)$ and the signal $S^{B}_{f_{P}}(t)$ averaged with respect to low-frequency peaks. For couplings based on non-linear phenomena or the cyclical occurrence in the signal of short-lived structures with a wide frequency band, we should observe modulations of energy density positioned in a certain range of low-frequency phases, and stretching over almost the whole range of analyzed frequencies. Also, the average signal should then have sharp edges, but with no clear high-frequency oscillations (compare Figs. 6b and 7h). It is worth noting that this type of information can not be obtained by observing band-pass filtered signals aligned to the low-frequency phase. In such a case, the sharp signal structures after convolution with the impulse response function of the filter would look like oscillations in the tested frequency band. In the case of a non-sinusoidal waveform, one can also expect the appearance of a structure corresponding to harmonic relations in the averaged spectrum $AS_{f_{P}}(f)$ and spectrum of the averaged signal $SA_{f_{P}}(f)$.
Testing for input-related non-stationarities: This recommendation is not directly supported in the eMI framework. A technique that gives some insight into the origin of the observed PAC, in the case of the event-related design, was proposed in Voytek et al. (2013). It should work reliably, especially if the analyzed effects conform to the model “signal plus ongoing random activity”. Otherwise, a more reliable interpretation can be made by comparison of jitters of the maximum of a high-frequency burst relative to stimulus, and relative to the predefined phase of the low-frequency oscillation as proposed in the Aru et al. (2015).
Temporal structure: The eMI methodology supports visual inspection of the average relation between high-frequency power augmentation and the low-frequency oscillation ($M^{B}_{f_{P}}(t,f)$). The averaged fragments of maps encompass three cycles of low-frequency. For a larger time scale, the signal can be processed in consecutive epochs. According to the results presented in “The influence of signal properties on PAC detection” a 3 s epoch is enough for eMI to detect coupling. The summarized results (as in Fig. 11) provide the possibility of a comprehensive interpretation.
Surrogates: The generation of surrogate data implemented in the proposed framework destroys the phase relation between the high and low-frequency components but preserves all the time-frequency structure of the process under investigation as described in “Surrogate data for eMI”. Additionally, it is suitable for both continuous and event-related experimental designs.
Specificity of the effects: This issue in Aru et al. (2015) is stated in terms of the evaluation of differences in CFC measures across conditions. We want to add an argument here that besides power stratification, possible differences in the precision of phase-locking and the span of the phase range of the power augmentation should be considered. For instance, in the light of Lisman’s (Lisman 2005) model, it could be expected that the larger number of items to store in sequential memory could correspond to a broader range of low-frequency phase with the augmented high-frequency power. Our polar phase-histogram provides this information, which is lacking in standard comodulograms.

### Limitations

The eMI method presented in this study has some potential limitations. Phase-amplitude coupling theoretically may exhibit not only in the form of the phase-dependent amplitude augmentation but also attenuation. We designed the eMI only for analysis of the augmentations. This is because we consider that it is more plausible physiologically, that a high-frequency signal appears as bursts of activity in some moments when the low-frequency phase is permitting, rather than there is a persistent high-frequency activity which is suppressed in some phase of low-frequency oscillation.

The other important remark is that the automatic assignment of coupling origins is not infallible. As we can see in Figs. 4 and 5 (bar plots presenting proportion of detections labeled as *Reliable/Ambiguous*) and Fig. 8 there are some cases in which the coupling origins are incorrectly assessed. That is why we strongly suggest double-checking this automatic process by visual inspection of auxiliary plots.

## Conclusions

In this study, we have developed, tested, and validated the eMI toolbox for calculating PAC, and compared it against two benchmark methods. The results described above indicate that the eMI is comparable with dPAC and MI in terms of sensitivity. However, it proved to be more selective in the dimension of frequency for phase.

Importantly, eMI additionally provides an automatic assessment of coupling origins, the issue which the reference methods do not address. Moreover, it solves most of the problems related to the interpretation of PAC raised in recent literature, and it offers a compact form of auxiliary plots and polar phase-histograms, which provide the further support in understanding and visual investigation of observed coupling.

## Footnotes

1 using MatlabⓇ routine *pwelch.m* with the hamming window of 2 seconds duration and vector of frequencies ranging from 1 to Nyquist frequency with 0.5 Hz step

2 MatlabⓇ routine *pchip.m*

3 with MatlabⓇ routine *filtfilt.m*

4 attenuation of 3dB on the edges of the band; designed using the *fdesign.m* routine

5 This deformation is implemented using MatlabⓇ routine *imresize.m*, which uses bicubic interpolation.

6 “*This coupling is too close to the lower frequency for amplitude limit, thus it is labeled as Ambiguous. If you want to examine it, decrease the lower limit of frequency for amplitude”.*

7 we cite after the authors: “The study was approved, and subjects gave written informed consent approved by the UC Berkeley Committee on Human Research, and the Stanford Institutional Review Board. Both datasets were recorded with a Tucker-Davis Technologies (TDT) recording system at a sampling rate of 3051.76Hz. After acquisition, data were re-referenced to the common average, resampled to 1000Hz, and high-pass filtered at 0.5Hz. The electrodes selected for this study did not show epileptic activity.”

8 The minimal value before and after normalization was 0.
